# Transcriptome and phenotyping analyses support a role for chloroplast sigma factor 2 in red‐light‐dependent regulation of growth, stress, and photosynthesis

**DOI:** 10.1002/pld3.43

**Published:** 2018-02-19

**Authors:** Sookyung Oh, Deserah D. Strand, David M. Kramer, Jin Chen, Beronda L. Montgomery

**Affiliations:** ^1^ Department of Energy – Plant Research Laboratory Michigan State University East Lansing MI USA; ^2^ Department of Biochemistry and Molecular Biology Michigan State University East Lansing MI USA; ^3^ UK Medical Center MN 150 University of Kentucky College of Medicine Lexington KY USA; ^4^ Department of Microbiology & Molecular Genetics Michigan State University East Lansing MI USA; ^5^ Present address: Max‐Planck‐Institut für Molekulare Pflanzenphysiologie Potsdam‐Golm Germany

**Keywords:** light signaling, photomorphogenesis, photosynthesis, plastid development, sigma factors, stress

## Abstract

Sigma factor (SIG) proteins contribute to promoter specificity of the plastid‐encoded RNA polymerase during chloroplast genome transcription. All six members of the SIG family, that is, SIG1–SIG6, are nuclear‐encoded proteins targeted to chloroplasts. Sigma factor 2 (SIG2) is a phytochrome‐regulated protein important for stoichiometric control of the expression of plastid‐ and nuclear‐encoded genes that impact plastid development and plant growth and development. Among SIG factors, SIG2 is required not only for transcription of chloroplast genes (i.e., anterograde signaling), but also impacts nuclear‐encoded, photosynthesis‐related, and light signaling‐related genes (i.e., retrograde signaling) in response to plastid functional status. Although SIG2 is involved in photomorphogenesis in Arabidopsis, the molecular bases for its role in light signaling that impacts photomorphogenesis and aspects of photosynthesis have only recently begun to be investigated. Previously, we reported that SIG2 is necessary for phytochrome‐mediated photomorphogenesis specifically under red (R) and far‐red light, thereby suggesting a link between phytochromes and nuclear‐encoded SIG2 in light signaling. To explore transcriptional roles of SIG2 in R‐dependent growth and development, we performed RNA sequencing analysis to compare gene expression in *sig2‐2* mutant and Col‐0 wild‐type seedlings at two developmental stages (1‐ and 7‐day). We identified a subset of misregulated genes involved in growth, hormonal cross talk, stress responses, and photosynthesis. To investigate the functional relevance of these gene expression analyses, we performed several comparative phenotyping tests. In these analyses, strong *sig2* mutants showed insensitivity to bioactive GA_3_, high intracellular levels of hydrogen peroxide (H_2_O_2_) indicative of a stress response, and specific defects in photosynthesis, including elevated levels of cyclic electron flow (CEF) and nonphotochemical quenching (NPQ). We demonstrated that SIG2 regulates a broader range of physiological responses at the molecular level than previously reported, with specific roles in red‐light‐mediated photomorphogenesis.

## 1 INTRODUCTION

Functional chloroplasts contain both nuclear‐ and chloroplast‐encoded proteins. Thus, light‐dependent plastid development depends on coordinate control and stoichiometric expression of nuclear and plastid genes. Sigma factor protein 2 (SIG2) is a member of the six‐member SIG family (i.e., SIG1–SIG6) in Arabidopsis that functions in chloroplasts as a subunit of the plastid‐encoded RNA polymerase (PEP) to regulate expression of chloroplast genes for chloroplast development and chlorophyll biosynthesis, which are central for photosynthesis (Kanamaru et al., 1999). Like other members of the SIG family, SIG2 exhibits sequence similarity to the prokaryotic sigma factor σ70 (Nagashima et al., 2004). Each sigma factor exhibits specificity in interacting with the promoters of its target gene(s) in chloroplasts and plays a distinctive role during growth and development (Yagi & Shiina, 2014). For example, both SIG2 and SIG6 are induced by light and are critical for chloroplast development and chlorophyll biosynthesis, as *sig2* and *sig6* mutant seedlings are pale to yellow‐green with underdeveloped chloroplasts (Ishizaki et al., 2005; Shirano et al., 2000). Additionally, SIG5 has been recognized as a stress‐responsive factor due to the observed rapid induction of this gene upon salt, cold, and high‐light stresses, as well as high sensitivity in the presence of salt and low levels of maximal quantum yield of PSII (Fv/Fm) observed in *sig5* mutants (Nagashima et al., 2004; Tsunoyama, Morikawa, Shiina, & Toyoshima, 2002). In spite of the sequence homology between SIG2 and SIG5, the role of SIG2 in stress responses has not been fully explored. Notably, *SIG2* is distinct from *SIG5* in that it does not exhibit a transcriptional change upon specific experimental stress treatments, such as high light or salt (Nagashima et al., 2004).

Sigma factor protein 2 has been previously shown to have roles in both forward signaling from the nucleus to chloroplasts (i.e., anterograde signaling) that controls the transcription of chloroplast genes during photomorphogenesis (Oh & Montgomery, 2013), as well as in signaling from plastids back to the nucleus, that is, retrograde signaling, to control nuclear gene expression in response to plastid functional status (Woodson, Perez‐Ruiz, Schmitz, Ecker, & Chory, 2013). During anterograde signaling, phytochromes regulate expression of *SIG* genes, including *SIG2,* in a process required for promoting chloroplast development and function (Oh & Montgomery, 2013, 2014). During anterograde signaling, SIG2 contributes to regulating the expression of a number of plastid genes, including *psaJ*,* psbA*,* psbD*,* psbB*,* psbN*,* trnE*, and *trnV* (Oh & Montgomery, 2013; Woodson et al., 2013). During retrograde signaling, SIG2 is involved in controlling the transcription of a small set of photosynthetic associated nuclear genes (PhANGs), including *RBCS2B* (*Rubisco small subunit*), *LHCB1.2* (*LIGHT‐HARVESTING CHLOROPHYLL B‐BINDING 1*), *LHCB2.2* (*LIGHT‐HARVESTING CHLOROPHYLL B‐BINDING 2*), *PC* (*PLASTOCYANIN*), and *CA* (*CARBONIC ANHYDRASE*) (Woodson et al., 2013). In addition to SIG2, heme derivatives, including intermediates in chlorophyll biosynthesis, reactive oxygen species (ROS), and hormones, can initiate retrograde signaling (reviewed in Leister, 2012).

SIG2 has a recognized role in light‐dependent aspects of plant development, including the aforementioned chloroplast development and in distinct aspects of photomorphogenesis. Related to its role in chloroplast development, *sig2* mutants are chlorophyll deficient in white light (Oh & Montgomery, 2013; Shirano et al., 2000; Woodson et al., 2013), and in red and blue light (Oh & Montgomery, 2013). In regards to SIG2‐dependent regulation of photomorphogenesis in a wavelength‐specific manner, we noted that disruption of *SIG2* resulted in red (R)‐ and far‐red (FR)‐light‐specific defects in the inhibition of hypocotyl elongation and cotyledon expansion (Oh & Montgomery, 2013). Root elongation also was disrupted under R illumination in *sig2* mutants (Oh & Montgomery, 2013). However, the molecular bases of SIG2‐mediated, R‐, and/or FR light‐specific signaling that impacts photomorphogenesis and photosynthesis are not fully understood.

To investigate the role of SIG2 in regulating expression of genes in the chloroplast and nucleus at the molecular level, several transcriptional profiling analyses have been performed (Nagashima et al., 2004; Woodson et al., 2013). DNA microarray analysis shows that several chloroplast genes, including *psaJ*, which is transcribed by PEP, and additional plastid genes transcribed by the nuclear‐encoded, chloroplast‐localized RNA polymerase (NEP), are misregulated in a *sig2‐1* T‐DNA mutant (Nagashima et al., 2004). RNA‐Seq and microarray analyses using white‐light‐grown *sig2‐2* mutant seedlings resulted in the identification of a role for SIG2‐mediated retrograde signaling in the regulation of PhANGs (Woodson et al., 2013). Here, to assess the transcriptional role of SIG2 in its recently identified role in R‐dependent photomorphogenesis, plant development, and regulation of photosynthesis, we performed RNA‐Seq analysis to compare gene expression in a *sig2‐2* mutant and wild‐type (WT) seedlings grown in R light at two developmental stages (i.e., at 1 and 7 days). Our data suggested that, in red light, SIG2 is involved in anterograde‐ and retrograde‐dependent transcriptional regulation of growth‐, hormone‐, stress‐, and photosynthesis‐related genes. To investigate the functional relevance of these gene expression analyses to the observed red‐light, SIG2‐dependent aspects of growth and development, we conducted a battery of phenotyping tests and determined that *sig2* mutants show insensitivity to GA, high intracellular levels of hydrogen peroxide (H_2_O_2_) that are indicative of stress, and specific defects in photosynthetic performance, such as elevated levels of cyclic electron flow (CEF) and NPQ. These results implicate SIG2 in regulating a broader range of light‐dependent physiological responses at the molecular level than previously recognized.

## 2 EXPERIMENTAL PROCEDURES

### 2.1 Plant material

*Arabidopsis thaliana* Col‐0 ecotype was used as wild‐type (WT), and T‐DNA insertion mutant lines *sig2‐2* (SALK_045706), *sig2‐3* (SALK_022546), and transgenic line *sig2‐4* were previously described (Oh & Montgomery, 2013).

### 2.2 Plant growth conditions

For RNA sequencing (RNA‐Seq) analyses, Arabidopsis seeds were surface sterilized prior to sowing. In detail, seeds were incubated with 35% (v/v) commercial bleach containing 0.025% (v/v) SDS for 15 min, and then seeds were rinsed with sterile distilled water five times and planted on growth medium containing 1× Murashige and Skoog (MS) salts (Caisson Laboratories), 1% (w/v) sucrose, and 0.7% (w/v) Phytoblend (Caisson Laboratories). Seeds on solid medium were stratified for 4 days at 4°C in the dark and were then incubated in a Percival LED (light‐emitting diode)‐equipped growth chamber (Model: E‐30LED, Serial Number: 6447.02.04J) with red (R) LEDs (λ_max_ ~670 nm) under constant red (Rc) illumination for 1 day or 7 days at 22°C. For photosynthesis experiments, Arabidopsis seeds without sterilization were cold‐stratified for 4 days at 4°C in the dark and were grown on soil in controlled growth chamber (Conviron) at 22°C under long‐day condition (8 hr dark/16 hr light cycle) with cool‐white light lamps (F96T12 CW VHO, Phillips). Fluence rates were measured using a LI‐250A Light Meter with a Quantum sensor (LI‐COR) in temperature‐ (i.e., 22°C) and humidity‐controlled growth chambers.

### 2.3 RNA extraction and RNA‐Seq analysis

Total RNA was isolated from 1‐ or 7‐d‐old whole seedlings grown in Rc illumination (50 μmol m^−2^ s^−1^) as described above using an RNeasy^®^ Plant Mini kit (Qiagen). Three biological replicates were prepared, and the quality of RNA from samples was assessed with a Bioanalyzer (Agilent) to identify samples with a RNA integrity score (>7.5). RNA‐Seq samples were prepared using the Illumina TruSeq mRNA prep kit, and RNA‐Seq was performed by the Research Technology Support Facility at Michigan State University using the Illumina sequencing system, which employs a massively parallel sequencing‐by‐synthesis four‐dye approach to generate billions of bases of high‐quality DNA sequence per run (Mardis, 2008) and yields >120 million reads of 55 nucleotides in length. We employed cufflink and cuffdiff (Trapnell et al., 2012) to estimate variance–mean dependence in count data from high‐throughput sequencing assays and tested for differential expression based on a model using the negative binomial distribution. RNA‐Seq data have been deposited to the NCBI Gene Expression Omnibus database (submission number GSE104518).

### 2.4 Validation of RNA‐Seq data

To validate the RNA‐Seq data, quantitative RT‐PCR (qRT‐PCR) analyses for indicated genes (primers used are listed in Table S1) were performed with three biological replicates as described previously (Oh, Warnasooriya, & Montgomery, 2013). *UBC21* (*At5g25760*) was used as an internal control gene for normalization in the qRT‐PCR experiments.

### 2.5 Effect of gibberellic acid (GA_3_) or GA inhibitor Paclobutrazol (PAC) on hypocotyl development

To assess the effect of exogenous gibberellic acid (GA_3_; Sigma‐Aldrich) and its inhibitor paclobutrazol (PAC; Sigma‐Aldrich) on hypocotyl development of *sig2* mutants in Rc light, we performed light‐dependent hypocotyl length assays as described in Warnasooriya and Montgomery (2009). Briefly, seeds were planted on MS salt plates containing various concentrations of GA_3_ or PAC and cultured in a growth chamber with Rc light at 50 μmol m^−2^ s^−1^ for 7 days at 22°C.

### 2.6 Measurement of hydrogen peroxide (H_2_O_2_)

Measurement of intracellular hydrogen peroxide (H_2_O_2_) levels was performed as described in Strand et al. (2015). In brief, leaf disks from 40‐d‐old plants grown on soil at 22°C under white light with a long‐day photoperiod, or 7‐d‐old seedlings grown on MS medium under continuous white light, were harvested, with four biological replicates assessed per genotype. Leaf disks or whole seedlings were ground using liquid nitrogen and extracted in 50 mM potassium phosphate buffer (pH 7.2). After centrifugation of the extract, 10 μl of the resulting supernatant was incubated with Amplex red (Invitrogen, final concentration of 50 μM) and 0.2 units ml^−1^ horseradish peroxidase in the dark for 30 min at room temperature. The level of hydrogen peroxide was estimated using excitation at 530 nm and emission at 590 nm. The pellets remaining after centrifugation of leaf disk or whole seedling extracts were used to extract chlorophylls with 80% (v/v) of acetone and chlorophyll concentrations determined by equations described in Inskeep and Bloom (1985). Hydrogen peroxide measurements were normalized to total chlorophyll content.

### 2.7 Chlorophyll fluorescence and photosynthetic parameter measurements

Chlorophyll fluorescence data for 2‐week‐old plants grown at 22°C under white light (approximately 100 μmol m^−2^ s^−1^) with a long‐day photoperiod (16 hr light and 8 hr dark cycle) were obtained using a custom‐designed plant imaging system described previously (Cruz et al., 2016), with a few modifications as detailed below. Two‐week‐old plants were treated with five different light regimes (R1 to R5) of 24 hr each for 5 days under a 16 hr light and 8 hr dark cycle. Under the first light regime (R1), the plants were grown at constant light intensity of 100 μmol m^−2^ s^−1^ during the light period. For the second light regime (R2), plants were treated with sinusoidal light with a maximum light intensity of 500 μmol m^−2^ s^−1^ in the light phase. For the third light regime (R3), fluctuating sinusoidal light with a maximum intensity of 1,000 μmol m^−2^ s^−1^ was applied. For the fourth (R4) and fifth (R5) regimes, additional R1 and R3 treatments were reapplied, respectively.

For in vivo spectroscopic assays using 40‐d‐old plants grown at 22°C under white light (approximately 100 μmol m^−2^ s^−1^) with a long‐day photoperiod, chlorophyll fluorescence was measured as previously described (Avenson, Cruz, & Kramer, 2004; Ioannidis, Cruz, Kotzabasis, & Kramer, 2012). In detail, attached, fully expanded rosette leaves from dark‐adapted adult plants were used for experiments. Chlorophyll fluorescence was measured after irradiation with actinic light (116‐500 μmol m^−2^ s^−1^) using an in‐house‐made spectrophotometer (Hall et al., 2013; Livingston, Cruz, Kohzuma, Dhingra, & Kramer, 2010), and chlorophyll fluorescence yields (e.g., F_0_, F_M_, F_S_, F_M′_, and F_M″_) were estimated as described (Baker, Harbinson, & Kramer, 2007). Quantum yield of PSII (Φ_II_), nonphotochemical quenching (NPQ), and linear electron flow (LEF) were calculated using the chlorophyll fluorescence yield values as described in Strand et al. (2015). Electrochromic shift (ECS) measurements for analysis of proton motif force (*pmf*) were performed using dark‐interval relaxation kinetics (DIRK) spectroscopy, and ECS parameters were calculated as described in Baker et al. (2007) and Strand et al. (2015). Cyclic electron flow (CEF) was estimated by comparing the slope of the line obtained from graphing $v_{H}^{+}$ (light‐driven proton flux) vs. LEF (linear electron flow) for a particular line to the slope obtained for wild‐type (Avenson et al., 2004) and was normalized by chlorophyll per leaf area. Chlorophyll was extracted directly from ground leaf tissue with 80% (v/v) acetone and quantified according to Inskeep and Bloom (1985).

### 2.8 SDS‐PAGE gel and immunoblot analysis

For SDS‐polyacrylamide gel electrophoresis (SDS‐PAGE), total proteins were extracted from liquid‐nitrogen‐ground leaves of 40‐d‐old plants grown in white light (approximately 100 μmol m^−2^ s^−1^) with a long‐day photoperiod using extraction buffer (10 mM Tris‐HCl, pH 7.2). Proteins were resolved on 15% (w/v) SDS‐PAGE gel and immunoblotted to polyvinylidene difluoride (PVDF) membrane as previously described (Montgomery, Yeh, Crepeau, & Lagarias, 1999) with minor modifications. The membrane was blocked with 2% (w/v) bovine serum albumin (BSA) in tris‐buffered saline and incubated with 1:4,000 dilution of anti‐NDH18 antibody (Peng, Fukao, Fujiwara, Takami, & Shikanai, 2009), followed by horseradish peroxidase‐conjugated anti‐rabbit IgG (Pierce 1858415; Lot No. HJ108849; 1:4,000 diluted). Protein signal was detected using WesternBright enhanced chemiluminescence (Advansta).

### 2.9 Statistical analyses

For statistical analysis, two‐tailed Student's *t* test analyses (unpaired with two‐sample equal variance) were performed with Microsoft Excel 2007 software.

## 3 RESULTS

### 3.1 SIG2 is required for the retrograde signaling‐dependent transcriptional regulation of a distinct group of nuclear genes, including growth‐, GA‐, stress‐, and photosynthesis‐related genes

**Figure 1:**
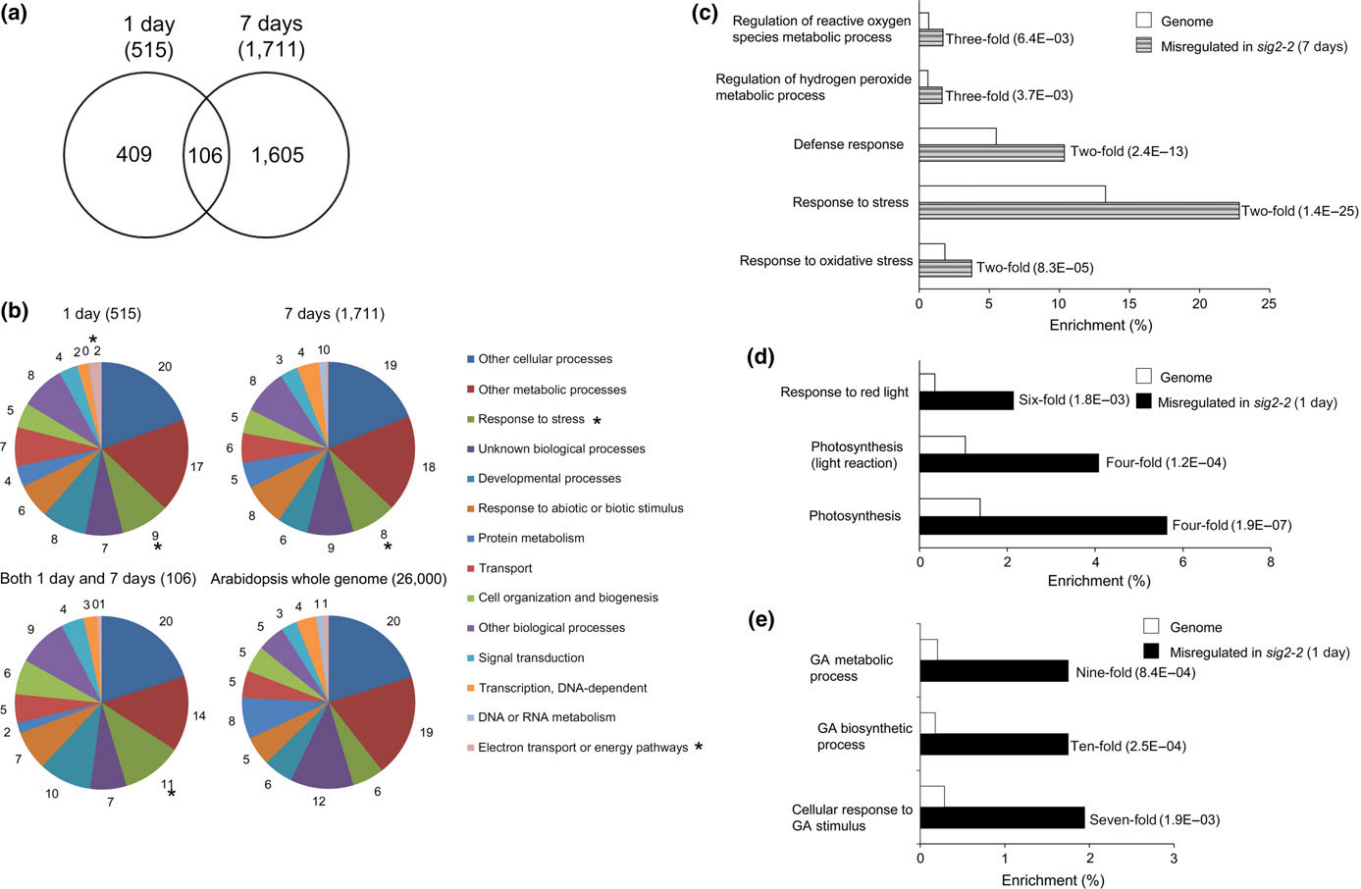
RNA‐Seq analysis of genes misregulated in *sig2‐2* mutant. (a) Venn diagram of genes misregulated in *sig2‐2* compared with Col‐0 wild‐type (WT) (≥2‐fold changes, *q*‐value <.05). One‐day‐old or seven‐day‐old seedlings grown on MS medium containing 1% sucrose and 0.7% Phytoblend agar at 22°C under Rc (50 μmol m^−2^ s^−1^) were used. (b) GO annotations of genes misregulated in *sig2‐2* using TAIR (www.arabidopsis.org/tools/bulk/go/index.jsp). Misregulated genes and ~26,000 genes from Arabidopsis whole genome were grouped based on GO categorization, and the enrichment of selected categories was indicated as percentage. An asterisk on the pie chart indicates a category of interest. (c to e) Categorization of genes misregulated in one‐day‐old *sig2‐2* (d, e) or seven‐day‐old *sig2‐2* (c) seedlings using Go::TermFinder. Enrichment of selected categories in GO biological process is indicated as fold change and *p* values (in parentheses) on bar graph

To understand the role of SIG2‐dependent transcriptional regulation that impacts photomorphogenesis and growth in Rc light, we performed RNA‐Seq analysis comparing gene expression changes between Col‐0 wild‐type (WT) and *sig2‐2* mutant lines at the whole genome level. Given the multiple morphological and chlorophyll pigmentation defects observed at distinct developmental stages for *sig2* mutant seedlings under R light (Oh & Montgomery, 2013), we used RNA extracted from *sig2‐2* mutant seedlings grown on MS medium under constant R light at 50 μmol m^−2^ s^−1^ for 1 or 7 days. From a RNA‐Seq‐based comparison of 1‐ or 7 days‐old samples of WT and *sig2‐2* mutant seedlings, we identified 515 and 1,711 misregulated genes (≥2‐fold changes, *q*‐value <.05), respectively (Figure 1a). Among these genes, 106 genes were misregulated at both 1 and 7 days, with 409 genes distinctly misregulated early in development at 1 day and 1,605 genes misregulated at 7 days. The misregulated genes were subjected to functional classification using gene ontology (GO)::TermFinder (Boyle et al., 2004) or (GO) annotations of TAIR (www.arabidopsis.org/tools/bulk/go/index.jsp). Among 515 genes misregulated in 1 day samples of *sig2‐2* mutant seedlings, 127 categories were significantly overrepresented in GO::TermFinder analysis (Table S2). By comparison, functional classification (Boyle et al., 2004) of genes misregulated in *sig2‐2* from 7 days using (GO)::TermFinder samples indicated that 255 categories were significantly overrepresented (Table S3).

During the growth and development of plants, expansion of the cell wall is essential. The cell wall is comprised of a complex network of various polysaccharides, including cellulose, hemicelluloses, and pectins, as well as glycoproteins (e.g., extensins) (Keegstra, 2010). Extensins are hydroxyproline‐rich glycoproteins (HRGPs), covalently cross‐linked in cell walls, and have been recognized as important proteins in the expansion of cell walls (Cannon et al., 2008; Lamport, Kieliszewski, Chen, & Cannon, 2011). Additionally, extensins are implicated in root development at distinct developmental stages (reviewed in Somssich, Khan, & Persson, 2016). Based on distinct repetitive amino acid sequence related to potential protein function, the 20 extensin proteins in Arabidopsis have been categorized into four distinctive groups, I, IIa, IIb, and IIc (Cannon et al., 2008). We noticed that all eight genes in group IIb (i.e., *EXT6* – *EXT13*) were highly upregulated in the *sig2‐2* mutant at 1 day, whereas these genes were all downregulated in the *sig2‐2* mutant at 7 days, compared to Col‐0 WT (Figure S1 and Table S4). We, then, generated heat maps for the expression of extensin genes in various tissues and light treatments using public Arabidopsis microarray database (AtGenExpress) and observed high levels of expression of these extensin genes in roots, suggesting root‐related functions (Figure S2). Notably, the *sig2‐2* mutant exhibits short roots in red‐light conditions (Oh & Montgomery, 2013). Relatedly, Velasquez et al. (2011) reported a short root hair phenotype for homozygous T‐DNA mutant lines for *EXT6*,* EXT7*,* EXT10*,* EXT11*, and *EXT12* type IIb extensin genes. Additionally, consistent with the previously observed disruption in the root elongation phenotype in *sig2‐2* (Oh & Montgomery, 2013), multiple genes related to root development or differentiation and root morphology were misregulated in this line under red light (Table S2).

A disproportionate number of misregulated genes in *sig2‐2* were assigned to the “response to stress” category, as compared to those in the whole genome of Arabidopsis (11% in “both 1 and 7 days” group of genes vs. 6% in Arabidopsis whole genome) (Figure 1b). Among these, subsets of genes associated with the regulation of reactive oxygen species (ROS) metabolic processes, regulation of hydrogen peroxide metabolic processes, response to oxidative stress, and responses to defense/stress were highly overrepresented (Figure 1c). These results suggested that regulation of stress‐related genes by SIG2‐mediated signaling is correlated with photomorphogenesis of Arabidopsis seedlings in Rc light.

A number of electron transport/energy pathway‐related genes were also overrepresented in a comparison of *sig2‐2* and WT at 1 day as compared with the whole genome of Arabidopsis (2% in 1 day of *sig2‐2* vs. WT compared to 1% in Arabidopsis whole genome) (Figure 1b). In addition, a number of nuclear photosynthesis‐related genes and R‐light‐responsive genes were misregulated in *sig2‐2* relative to WT, as compared to those in the entire genome of Arabidopsis (Figure 1d). This observation of misregulation of photosynthesis‐related genes under red‐light conditions is consistent with the recognized involvement of sigma factor proteins in transcriptional regulation of photosynthesis genes (Kanamaru et al., 1999) and its previously described role in white light as a retrograde signaling component for the regulation of PhANGs (Woodson et al., 2013).

A number of gibberellins (GA) biosynthetic process‐related genes were significantly (~10‐fold) misregulated in *sig2‐2* relative to WT, as compared to those in the entire genome of Arabidopsis (Figure 1e, Table S2). Genes related to GA metabolic processes (ninefold) and cellular responses to GA stimulus (sevenfold) were also significantly misregulated in the *sig2‐2* mutant (Figure 1e, Table S2). These observations suggest a role for SIG2‐mediated aspects of GA signaling, perhaps as a part of SIG2‐mediated retrograde signaling. As most hormones are synthesized at least partly in chloroplasts, these molecules can be considered as triggers for retrograde signaling. For example, exogenous treatment of plants with gibberellic acid (GA) results in the repression of both transcription (tested by run‐on assays) and transcript accumulation (tested by RNA blot hybridization) of chloroplast genes (Zubo, Yamburenko, Kusnetsov, & Börner, 2011). However, the precise role of GA in the SIG2‐mediated retrograde signaling has not been demonstrated.

### 3.2 Retrograde‐dependent role of SIG2 in GA signaling during photomorphogenesis

**Figure 2:**
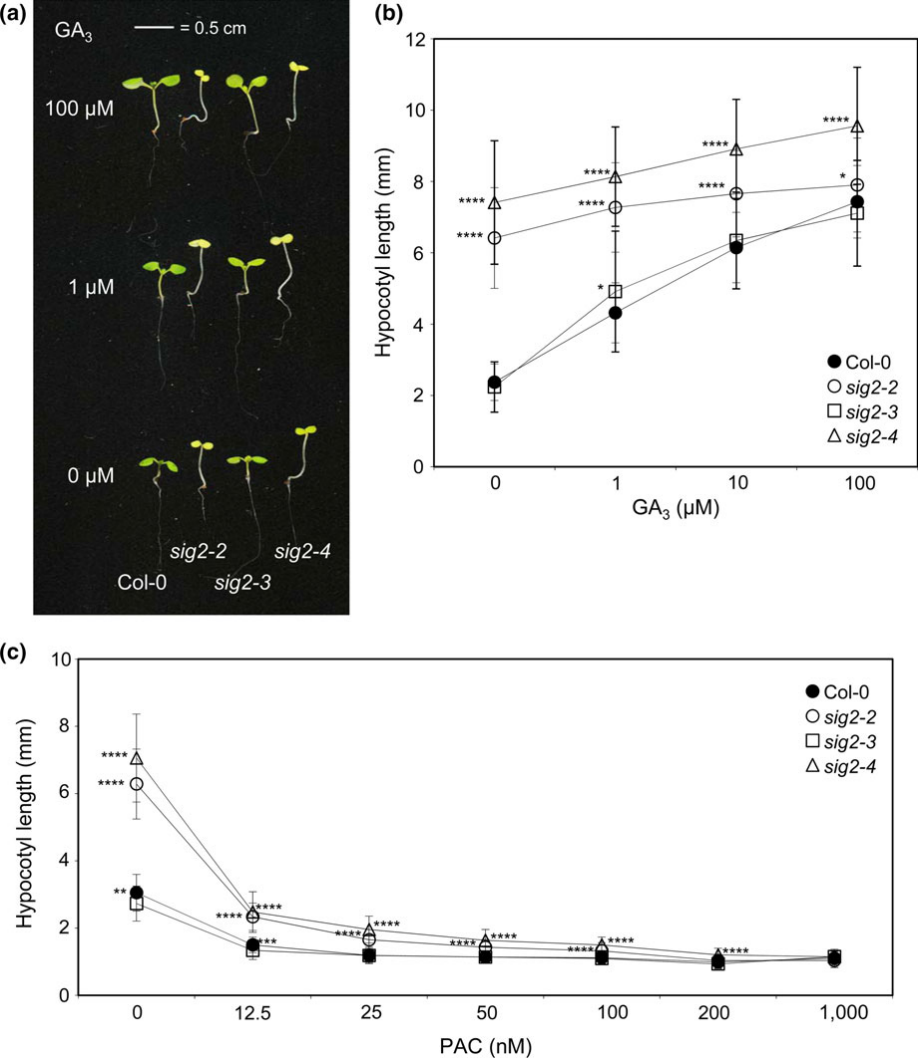
GA_3_‐sensitivity test on hypocotyl growth in Col‐0 WT and *sig2* mutant seedlings. Seedlings were grown on MS medium containing 1% sucrose and 0.7% Phytoblend agar with various concentrations of (a, b) gibberellic acid (GA_3_) or (c) paclobutrazol (PAC) at 22°C for 7 days under constant Rc (50 μmol m^−2^ s^−1^). (a) Representative images of seedlings were shown. The scale bar indicated 0.5 cm. (b, c) Data points in graphs represent mean hypocotyl lengths of seedlings (±*SD*,* n* ≥ 25). Unpaired, two‐tailed Student's *t* test comparing *sig2* mutants to Col‐0 WT for each (B) GA_3_ or (C) PAC concentration, *p < .05, **p < .005, ***p < .0005, ****p < .00005

Loss of function of *SIG2* causes photomorphological defects such as a long hypocotyl and small‐ and pale‐green cotyledons in R light (Oh & Montgomery, 2013). As *SIG2* is regulated by phytochrome and these phenotypic defects were specific to wavelengths associated with phytochrome signaling, these observed photomorphogenic phenotypes suggest a role of SIG2 in phytochrome‐mediated, R‐light‐specific regulation during photomorphogenesis (Oh & Montgomery, 2013). GAs, tetracyclic diterpenoid hormones, promote hypocotyl elongation, while paclobutrazol (PAC) as a GA biosynthesis inhibitor prevents this elongation (Collett, Harberd, & Leyser, 2000; Cowling, Kamiya, Seto, & Harberd, 1998). Additionally, GA has been implicated in repressing photomorphogenesis in darkness (Alabadí, Gil, Blázquez, & García‐Martínez, 2004). GA is involved in phytochrome‐mediated hypocotyl development by regulation of DELLA proteins, which serve as transcriptional regulators for photomorphogenesis (Achard et al., 2007; Hedden & Phillips, 2000). Our RNA‐Seq analysis showed that GA signaling‐related genes were overrepresented in 1‐d‐old *sig2‐2* seedlings vs. WT, as compared to representation in the whole genome (Figure 1e). Given the elongated hypocotyl of *sig2‐2* in Rc (Oh & Montgomery, 2013; Figure 2a) and the impact of GA levels on hypocotyl elongation (Alabadí et al., 2004), we hypothesized that misregulation of GA genes in *sig2* mutants may be directly related to the observed growth defects. Thus, to assess the physiological relevance of these gene expression analyses, we tested the effect of exogenous gibberellic acid (GA_3_) or GA inhibitor paclobutrazol (PAC) on hypocotyl elongation of *sig2* mutants in Rc light. Wild‐type and a weak *sig2* mutant, that is, *sig2‐3*, showed long hypocotyls in the presence of high concentrations of GA_3_; however, two strong *sig2* mutants, *sig2‐2* and *sig2‐4*, exhibited only a minor response to exogenous GA_3_ treatment in regards to hypocotyl elongation in Rc light (Figures 2a and b). Additionally, *sig2‐2* and *sig2‐4* mutants did not exhibit any significant change in the development of cotyledons upon GA_3_ treatment (Figure 2a). PAC inhibited elongation of hypocotyls in both WT, as expected (Feng et al., 2008), as well as in *sig2* mutants in a dosage‐dependent manner (Figure 2c). Our RNA‐Seq data for GA signaling‐related genes, altered *sig2* mutant sensitivity to GA_3_, and standard responsiveness to PAC in *sig2* mutants suggested a retrograde‐associated transcriptional role of SIG2 in GA‐dependent processes during photomorphogenesis. Among misregulated GA genes in *sig2‐2*, a GA biosynthesis gene, *GIBBERELLIN 3‐OXIDASE 1* (*GA3OX1*,* At1g15550*), which was significantly (3.2‐fold) downregulated in a 7‐d‐old *sig2‐2* mutant, was confirmed by semiquantitative RT‐PCR analysis (Figure S3). This gene is of particular interest as it is involved in the conversion of inactive GA precursors to bioactive GA molecules, GA4 and GA1 (Zhou, Yu, & Pharis, 2004); yet, *GA3OX1* is also downregulated when seeds are treated with exogenous GA (Thomas, Phillips, & Hedden, 1999). Thus, misregulation of *GA3OX1* in addition to downregulation of *GA2OX4* (Table S5), which encodes a protein involved in inactivation of bioactive GAs (Yamaguchi, 2006), potentially suggested altered intracellular GA homeostasis. The regulation of *GA3OX1* and *GA2OX4*, however, is more complex in that these are also GA‐responsive genes, which show downregulation and upregulation, respectively, upon treatment of seedling with bioactive GA (Thomas et al., 1999).

### 3.3 Retrograde‐associated transcriptional role of SIG2 in H_2_O_2_‐mediated stress response

**Figure 3:**
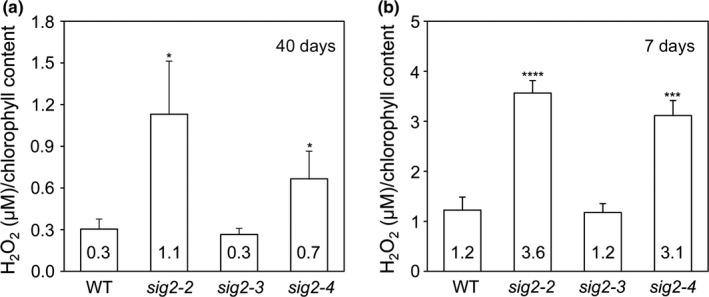
The measurement of H_2_O_2_ contents in wild‐type and *sig2* mutants. The level of H_2_O_2_ was measured using Amplex Red assay and normalized to total chlorophyll content. (a) The level of H_2_O_2_ measured using three leaf disks from wild‐type (WT) and *sig2* mutants grown on soil under white light (100 μmol m^−2^ s^−1^, long‐day condition with 8 hr dark/16 hr light cycle) at 22°C for 40 days. (b) The level of H_2_O_2_ measured using seedlings of WT and *sig2* mutants (*n* = 27) grown on MS medium under constant white light (100 μmol m^−2^ s^−1^) at 22°C for 7 days. Data points in graphs represent mean (±*SD*,* n* = 4 biological repeats). Unpaired, two‐tailed Student's *t* test comparing *sig2* mutants to Col‐0 WT, *p < .05, **p < .005, ***p < .0005, ****p < .00005

Functional categorization of misregulated genes from RNA‐Seq analysis showed that a large number of genes related to the regulation of reactive oxygen species (ROS) and hydrogen peroxide (H_2_O_2_) metabolic processes were misregulated in *sig2‐2* relative to WT (Figure 1c). To determine whether these gene expression changes were correlated with changes in intracellular ROS levels, we measured the level of H_2_O_2_ in *sig2* mutants grown in white light for either 7 or 40 days (Figure 3). The level of H_2_O_2_ was high (>2.3‐fold greater than WT) in *sig2‐2* or *sig2‐4* compared with WT, at both seedling and adult stages, but levels were not altered in weak mutant *sig2‐3* (Figure 3). Notably, ROS, intermediates from the Calvin–Benson–Bassham (CBB) cycle, ATP/ADP ratios, and NADPH/Fd redox states during photosynthesis, all have been proposed as potential signals for the regulation of cyclic electron flow (CEF), an important component in photosynthesis and which serves a role in protection from photostress (Breyton, Nandha, Johnson, Joliot, & Finazzi, 2006; Fan et al., 2007; Gambarova, 2008; Joliot & Joliot, 2002; Lascano, Casano, Martin, & Sabater, 2003; Strand et al., 2015). For example, infiltration of Arabidopsis leaves with H_2_O_2_ resulted in elevated levels of CEF, and CEF is indeed activated by H_2_O_2_ (Strand et al., 2015). Thus, the elevated H_2_O_2_ levels in strong *sig2* mutants may indicate disruptions in specific photosynthetic parameters.

### 3.4 SIG2 is required for the retrograde signaling‐dependent regulation of photosynthesis‐related genes in red light and maintenance of photosynthetic efficiency

As anticipated, given prior evidence of a role for SIG2 in chlorophyll biosynthesis and chloroplast development and signaling, we found that several nucleus‐encoded photosystem I (PSI)‐, photosystem II (PSII)‐related genes, and chlorophyll biosynthetic pathway genes were misregulated in *sig2‐2* (Table 1). We validated RNA‐Seq data of two nucleus‐encoded PSI/II‐associated genes, *LHCB2.4* (i.e., PSII gene) and *PsaE* (*photosystem I subunit E,* i.e., PSI gene) using quantitative RT‐PCR (qRT‐PCR) analysis (Figure S4). These two genes were chosen for validation of RNA‐Seq data because they were significantly downregulated in mesophyll‐specific phytochrome‐deficient lines, tested by our group previously (Oh et al., 2013), and, thus, may be related to the red‐light‐specific defects in *sig2‐2* mutants. Consistent with RNA‐Seq data, qRT‐PCR analysis demonstrated that *LHCB2*.4 was downregulated in *sig2‐2* (7 days), and *PsaE* was upregulated in *sig2‐2* (1 day). These results suggest that SIG2 has a role in transcriptional regulation of not only plastid‐encoded genes, but also nucleus‐encoded photosynthesis‐related genes, as previously reported for SIG2‐mediated retrograde signaling (Woodson et al., 2013). As similar expression patterns of several photosynthesis‐related genes were observed in mesophyll‐deficient phytochrome lines and *sig2* mutants, phytochrome‐dependent regulation of *SIG2* expression is important for anterograde signaling (Oh & Montgomery, 2013) and retrograde signaling back to the nucleus (Woodson et al., 2013).

**Table 1:** RNA‐Seq analysis on nucleus genes encoding subunits of photosystem I (PSI) and photosystem II (PSII) and encoding steps of the chlorophyll biosynthetic pathway

AGI no.	Gene ID	Function	1 day				7 days			
			WT	*sig2‐2*	*sig2‐2* /WT^a^	Cutoff^b^	WT	*sig2‐2*	*sig2‐2* /WT^a^	Cutoff^b^
*At3g61470*	*LHCA2*	PSI	18	37	1.0	Yes	2,241	1,869	−0.3	No
At1g61520*	*LHCA3*	PSI	39	84	1.1	Yes	3,841	3,633	−0.1	No
*At1g29920*	*LHCB1.1*	PSII	8	20	1.4	Yes	4,303	5,953	0.5	Yes
*At1g29930*	*LHCB1.3*	PSII	128	281	1.1	Yes	16,930	35,208	1.1	No
*At2g34430*	*LHCB1.4*	PSII	1	2	0.8	No	1,637	3,190	1.0	Yes
*At2g34420*	*LHCB1.5*	PSII	24	56	1.2	Yes	3,759	5,292	0.5	Yes
At3g27690*	*LHCB2.4*	PSII	1	1	−0.9	No	766	342	−1.2	Yes
*At5g54270*	*LHCB3*	PSII	35	73	1.1	Yes	2,773	2,570	−0.1	No
At5g01530*	*LHCB4.1*	PSII	59	119	1.0	Yes	2,643	2,319	−0.2	No
At2g40100*	*LHCB4.3*	PSII	4	7	0.6	No	26	5	−2.4	Yes
At4g10340*	*LHCB5*	PSII	48	93	1.0	Yes	3,510	2,915	−0.3	No
At2g20260*	*PsaE*	PSI	16	40	1.3	Yes	750	717	−0.1	No
At3g16140*	*PsaH1*	PSI	15	31	1.1	Yes	1,306	1,177	−0.1	No
At1g30380*	*PsaK*	PSI	11	37	1.8	Yes	2,799	2,050	−0.4	Yes
At4g12800*	*PsaL*	PSI	23	57	1.3	Yes	2,857	1,978	−0.5	Yes
At1g08380*	*PsaO*	PSI	14	28	1.0	Yes	3,624	2,607	−0.5	Yes
At4g05180*	*PsbQ‐2*	PSII	2	6	1.5	Yes	1,308	812	−0.7	Yes
At1g79040*	*PsbR*	PSII	59	126	1.1	Yes	5,064	4,319	−0.2	No
At5g54190*	*PORA*	CP	8	17	1.1	Yes	12	5	−1.2	Yes
At1g03630*	*PORC*	CP	69	80	0.2	No	196	82	−1.3	Yes

Genes were selected by >2‐fold change with significant cutoff (yes or no) in at least one sample between 1 and 7 days from RNA‐Seq analysis. Photosystem I (PSI) and PSII‐related genes, nuclear‐encoded; chlorophyll biosynthetic pathway genes (CP), nuclear‐encoded.

a Fold change (in log2 scale).

b Significant with *q*‐value <.05. Asterisks on AGI no. indicates genes, downregulated by retrograde signals as identified by microarray analysis from Tables S6–S7 of Woodson et al. (2013).

**Figure 4:**
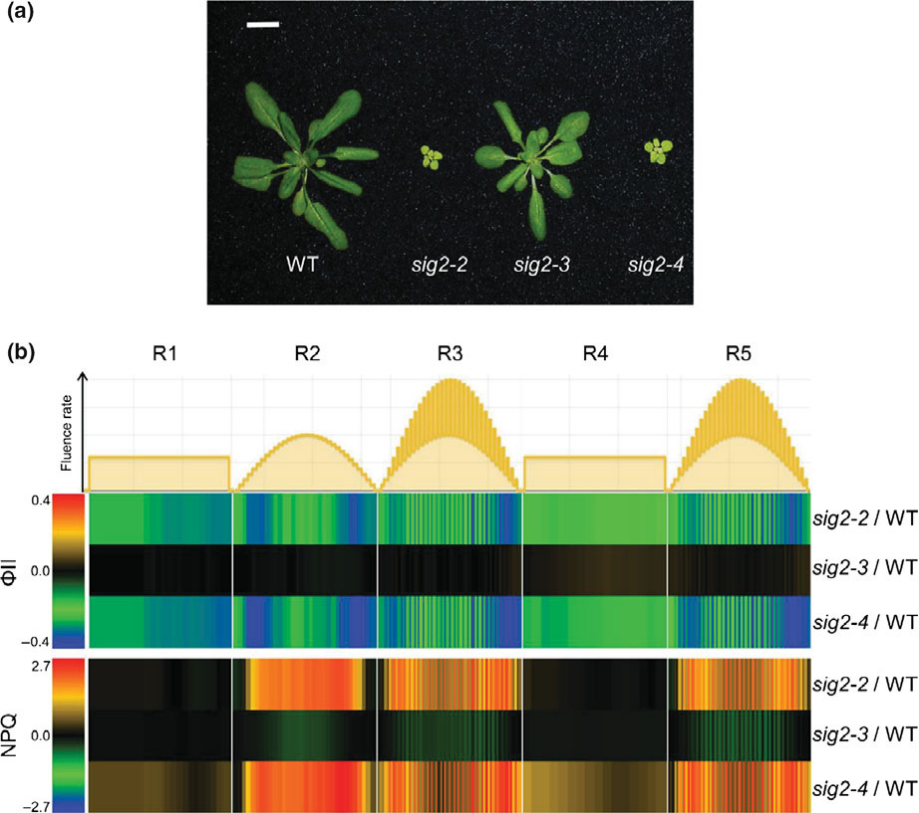
Φ_II_ and NPQ measurements in adult wild‐type and *sig2* mutants. (a) Representative image of adult plants grown on soil under white light (100 μmol m^−2^ s^−1^, long‐day condition with 8 hr dark/16 hr light cycle) at 22°C for 30 days. The scale bar indicated 1 cm. (b) Heatmap showing the level of Φ_II_ (photochemical efficiencies of PSII) and NPQ (nonphotochemical quenching), calculated from chlorophyll fluorescence images captured under five different light regimes (R1 to R5) during the course of 5 days using two‐week‐old plants grown on soil under white light (100 μmol m^−2^ s^−1^). R1, constant light (100 μmol m^−2^ s^−1^); R2, sinusoidal light with maximum 500 μmol m^−2^ s^−1^; R3, fluctuating light between two sinusoidal light intensity curves with maximum 1000 μmol m^−2^ s^−1^; R4 and R5, repeat R1 and R3, respectively. Difference between mutants and wild‐type (WT) was shown. Red denotes increased relative level of Φ_II_ or NPQ in mutants compared to WT; blue denotes decreased level of Φ_II_ or NPQ in mutants compared to WT

To evaluate the physiological impacts of apposite regulation of SIG2 and associated regulation of H_2_O_2_ levels on photosynthesis in vivo, we measured quantum efficiency of PSII (Φ_II_) and nonphotochemical quenching (NPQ) of *sig2* mutants compared to WT using a noninvasive, real‐time fluorescence imaging system as described previously (Cruz et al., 2016). At the adult stage, strong *sig2* mutants, *sig2‐2* or *sig2‐4*, were small and pale green with fewer leaves compared with a weak *sig2‐3* mutant or WT (Figure 4a), similar to phenotypes observed in seedlings (Oh & Montgomery, 2013; Shirano et al., 2000). Adult plants were treated with five different light regimes (R1 to R5) during a long‐day photoperiod of 16 hr light/8 hr dark for 5 days. In detail, R1 and R4 were constant laboratory light conditions at 100 μmol m^−2^ s^−1^ during the light period; R2 was sinusoidal light peaking at 500 μmol m^−2^ s^−1^ that was intended to mimic a sunny day; R3 and R5 represented cloudy days with fluctuating sinusoidal light with a maximum at 1,000 μmol m^−2^ s^−1^. *sig2‐2* and *sig2‐4* mutants exhibited low levels of Φ_II_ relative to WT, whereas *sig2‐*3 showed a level of Φ_II_ comparable to WT (Figure 4b). Additionally, the level of NPQ was high in *sig2‐2* and *sig2‐4* lines compared to WT (Figure 4b). Because plants were adapted under 100 μmol m^−2^ s^−1^ of white light before examining, the differences in photosynthetic parameters were most prominent when plants were exposed to higher or fluctuating light intensities (e.g., R2, R3, or R5).

### 3.5 Low photosynthetic performance and high rates of CEF are apparent in *sig2* mutants

**Figure 5:**
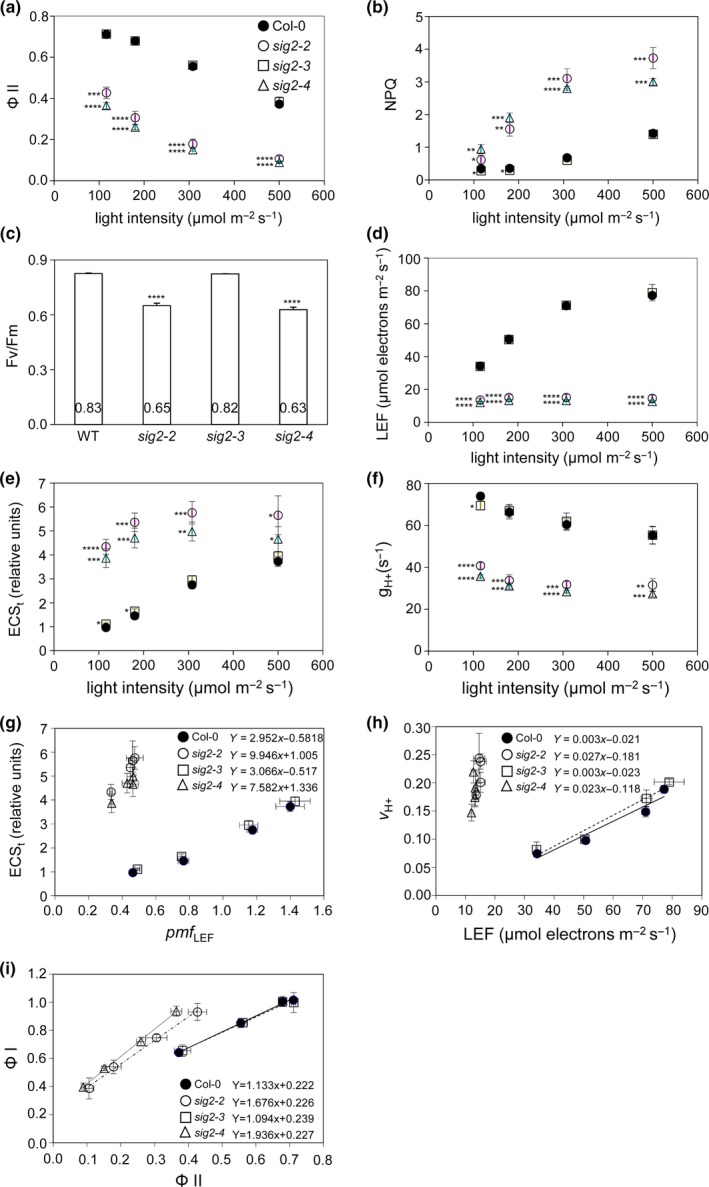
Measurement of photosynthetic properties in wild‐type and *sig2* mutants grown on soil. Plants were grown at 22°C under white light (100 μmol m^−2^ s^−1^, long‐day condition with 8 hr dark/16 hr light cycle) for 40 days. Undetached leaves from WT (filled circle), *sig2‐2* (open circle), *sig2‐3* (square), or *sig2‐4* (triangle) were used for measurements of distinct photosynthetic properties. (a) Φ_II_ (photochemical efficiency of PSII) vs. light intensity, (b) NPQ (nonphotochemical quenching) vs. light intensity, (c) maximal quantum yield of PSII (Fv/Fm), (d) LEF (linear electron flow) vs. light intensity, (e) ECS_t_ (total magnitude of ECS decay) vs. light intensity, (f) $g_{H}^{+}$ (steady‐state rate of proton flux) vs. light intensity, (g) ECS_t_ versus *pmf* _LEF_ (*pmf*, generated solely by LEF), (h) CEF (cyclic electron flow) determined from slope of the linear regression of $v_{H}^{+}$ vs. LEF, and (i) PSI (Φ_I_) vs. PSII (Φ_II_). Data points in graphs represent mean (±*SD*,* n* = 3 individual plants). (a–f) Unpaired, two‐tailed Student's *t* test comparing *sig2* mutants to Col‐0 WT, *p < .05, **p < .005, ***p < .0005, ****p < .00005. (G‐I) Linear regression equations are shown for each plant line

In addition to utilizing a real‐time fluorescence imaging system, we performed more detailed in vivo light‐induced fluorescence and absorbance spectroscopic assays using attached, expanded leaves from 40‐day‐old plants under various light intensities (116–500 μmol m^−2^ s^−1^) to examine more closely the effects of SIG2 on photosynthesis. Using saturation pulse chlorophyll fluorescence yield changes, we measured Φ_II_ and NPQ in WT and *sig2* mutants under these conditions. Similar to data obtained from a fluorescence imaging system (Figure 4b), the level of Φ_II_ was low (about twofold) in *sig2‐2* and *sig2‐4* (Figure 5a), and the level of NPQ was intensity‐dependent and about threefold higher in these mutant lines, compared with WT or *sig2‐3* (Figure 5b). *sig2‐2* and *sig2‐4* mutants exhibited lower (approximately 20% reduction) levels of maximal quantum yield of PSII (Fv/Fm), compared with WT (Figure 5c).

We also used Φ_II_ to estimate light‐saturated linear electron flow (LEF), which results in the synthesis of reducing power in the form of ATP and NADPH. By comparing relative estimates of proton flux with LEF, we estimated the rates of CEF, a process that is activated under environmental stresses and supplementing ATP pools to balance the ATP/NADPH ratio for photosynthesis and activate photoprotective NPQ (Strand et al., 2015). LEF was lower than WT in the *sig2‐2* and *sig2‐4* mutants, most prominently (fivefold lower than WT) under the highest light intensity tested (i.e., 500 μmol m^−2 ^s^−1^) (Figure 5d).

We also probed the extent of light‐induced thylakoid proton motive force (*pmf*) and proton flux through the ATP synthase using the dark‐interval relaxation kinetics (DIRK) of the electrochromic shift (ECS) as described previously (Cruz et al., 2005; Livingston et al., 2010; Sacksteder & Kramer, 2000). The light‐induced transthylakoid *pmf*, estimated by the ECS_t_ parameter, was high (>3‐fold higher) in *sig2‐2* and *sig2‐4* lines compared with WT (Figure 5e). The conductivity of the thylakoid membrane to proton efflux ($g_{H}^{+}$), which primarily indicates the activity of the chloroplast ATP synthase, was about twofold lower in *sig2* mutants compared with WT (Figure 5f). We estimated the *pmf* expected from LEF alone (*pmf*_LEF_), as described previously (Avenson et al., 2005). The slope of *pmf*_LEF_ vs. ECS_t_, used to estimate CEF, was high (approximately threefold more) in *sig2‐2* and *sig2‐4* mutants compared with WT (Figure 5g), suggesting that CEF was strongly activated in the mutants.

To investigate the impact of an alteration in SIG2 function on CEF more directly, we measured the relative light‐driven proton flux ($v_{H}^{+}$) using ECS techniques (Cruz et al., 2005; Sacksteder & Kramer, 2000) and estimated the level of CEF by comparing the slopes of $v_{H}^{+}$ relative to LEF in *sig2* mutants to WT. The steeper slope of $v_{H}^{+}$ vs. LEF in *sig2‐2* (slope equals to 0.027) and *sig2‐4* (slope equals to 0.023) mutants compared with WT (slope equals to 0.003) and *sig2‐3* (slope equals to 0.003) (Figure 5h) is consistent with strong activation of CEF. When the average of $v_{H}^{+}$/LEF from four light intensity experiments (116–500 μmol m^−2^ s^−1^) was calculated, *sig2‐2* and *sig2‐4* mutants exhibited high fold change (6.8‐fold, 6.6‐fold, respectively) in comparison with WT (Table 2). These results confirmed high CEF (as estimated above by plotting *pmf*_LEF_ vs. ECS_t_) in strong *sig2* mutants (Figure 5h).

**Table 2:** High CEF ($v_{H}^{+}$/LEF) in *sig2* mutants

Plants	Average	Standard deviation	Fold change (vs. Col‐0 WT)
Col‐0	0.0022	0.0002	1.0
*sig2‐2*	0.0148	0.0018	6.8
*sig2‐3*	0.0023	0.0002	1.1
*sig2‐4*	0.0143	0.0022	6.6

CEF ($v_{H}^{+}$/LEF) values from four light intensity experiments (116–500 μmol m^−2^ s^−1^) in plants were averaged, and fold change was calculated.

Cyclic electron flow involves PSI‐dependent recycling of electrons from reduced ferredoxin of NADPH to the cytochrome *b*_6_ *f* complex via plastocyanin. Thus, high CEF mutants often exhibit high PSI quantum efficiencies (Φ_I_) (Livingston et al., 2010). Φ_I_ was estimated as described previously (Klughammer & Schreiber, 1993) and compared with Φ_II_ (Figure 5i). The slope of Φ_I_ vs. Φ_II_ was higher for *sig2‐2* and *sig2‐4* mutants than WT or the weak *sig2‐3* mutant (slope of linear regression of *sig2‐2*: 1.68; *sig2‐4*: 1.94; WT: 1.14; *sig2‐3*: 1.09), indicating higher efficiency of Φ_I_ than that of Φ_II_ in *sig2* mutants. These data further supported high PSI‐related levels of CEF in *sig2* mutants.

### 3.6 CEF‐impacting components in *sig2* mutants

**Figure 6:**
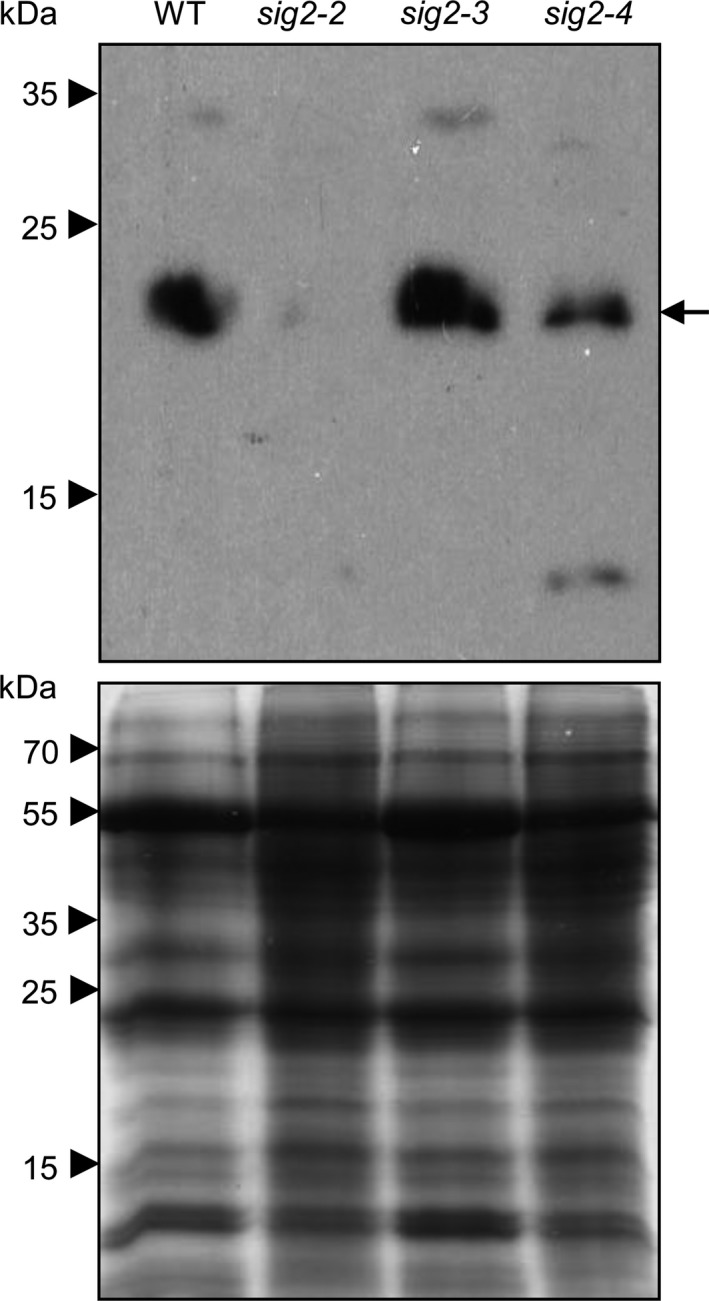
Accumulation of NDH18 proteins in wild‐type and *sig2* mutants. Western blot analysis was performed using anti‐NDH18 antibody (Top panel). Total soluble proteins were extracted from rosette leaves of 40‐day‐old plants grown on soil at 22°C under white light (100 μmol m^−2^ s^−1^, long‐day condition with 8 hr dark/16 hr light cycle), and were resolved on 15% SDS‐PAGE gel (Bottom panel). An arrow indicates NDH18 proteins (estimated as 23.8 kDa)

The ~550 kDa NADPH dehydrogenase supercomplex (NDH), responsible for one of the proposed CEF pathways (Burrows, Sazanov, Svab, Maliga, & Nixon, 1998; Endo, Shikanai, Sato, & Asada, 1998), consists of more than 31 subunits in Arabidopsis (Peng, Yamamoto, & Shikanai, 2011; Suorsa, Sirpiö, & Aro, 2009) and is similar to the photosystems in that it consists of both nuclear‐ and plastid‐encoded proteins (Rumeau et al., 2005; Suorsa et al., 2009). Among components of NDH complex, 11 subunits (NDH‐A to K) are encoded by chloroplast genes, and more than 20 of the subunits are encoded by nuclear genes (Peng et al., 2011; Suorsa et al., 2009). Based on the localization of the protein complex on thylakoid membranes and interactions with other partner proteins, the NDH complex could be divided into four subcomplexes, membrane subcomplex, subcomplex A, subcomplex B, and lumen subcomplex (Peng et al., 2009). We noticed that several nuclear NDH subunit genes (*NDHM*,* NDHN*,* CRR3*,* CRR7*,* NDH48*/*NDF1*,* NDH45*/*NDF2*,* NDF6*,* NDH18*,* CRR6*/*NDF3*,* NDF5*,* TLP21*,* FKBP16‐2*, and *PsbQ‐like*) were moderately misregulated in *sig2‐2* mutant grown in Rc light for 7 days (Table 3). Among them, the *NDH18* gene, encoding a component of subcomplex B, was downregulated (1.6‐fold) in the *sig2‐2* mutant at 7 days (Table 3). Protein gel blot analysis using extracts from 40‐day‐old plants showed less accumulation of NDH18 in *sig2‐2* and *sig2‐4*, compared with WT or *sig2‐3*, indicating that the downregulation of *NDH18* gene correlated with reduced protein accumulation in *sig2‐2* and *sig2‐4* mutants (Figure 6). Previously, it also has been proposed that ferredoxin quinone reductase complex (FQR) is involved in the regulation of CEF (Bendall & Manasse, 1995) and that Proton Gradient Regulation 5 (PGR5) and PGR5‐like protein 1 (PGRL1) might be components of this complex (DalCorso et al., 2008; Munekage et al., 2002). Our RNA‐Seq analysis showed that the expression of genes encoding *PGR5* and *PGRL1* was unchanged in *sig2‐2* mutant seedlings compared to WT (Table 3).

**Table 3:** Expression of genes encoding NDH subunits, auxiliary proteins for the NDH complex, and PGR proteins

Gene name	1 day				7 days			
	WT	*sig2‐2*	*sig2‐2* /WT^a^	Cutoff^b^	WT	*sig2‐2*	*sig2‐2* /WT^a^	Cutoff^b^
*NDHM*	5	6	0.18	No	126	90	−0.47	Yes
*NDHN*	13	18	0.47	Yes	134	97	−0.46	Yes
*NDHO*	15	29	0.90	Yes	183	165	−0.15	No
*CRR23/NDHL*	13	21	0.70	No	302	252	−0.26	No
*CRR3*	3	6	1.02	Yes	47	36	−0.39	Yes
*CRR7*	25	32	0.38	No	49	39	−0.32	Yes
*NDH48/NDF1*	8	10	0.35	No	146	119	−0.29	Yes
*NDH45/NDF2*	29	45	0.65	Yes	154	117	−0.40	Yes
*NDF6*	21	31	0.57	Yes	350	280	−0.32	Yes
*NDH18*	25	38	0.58	Yes	196	120	−0.71	Yes
*CRR1*	20	25	0.32	No	48	43	−0.14	No
*CRR2*	20	20	−0.06	No	9	10	0.05	No
*CRR4*	1	1	0.28	No	1	1	−0.23	No
*CRR6/NDF3*	30	39	0.37	Yes	55	67	0.28	Yes
*CRR21*	10	13	0.40	Yes	14	14	0.07	No
*CRR22*	6	5	−0.11	No	5	5	0.02	No
*CRR28*	7	10	0.47	No	6	6	0.20	No
*CP31A*	622	720	0.21	No	675	590	−0.19	No
*PGR3*	17	20	0.23	No	19	22	0.23	No
*NDF4*	23	29	0.37	No	117	75	−0.65	No
*NDF5*	14	17	0.28	No	26	21	−0.28	Yes
*PIFI*	12	15	0.35	No	140	125	−0.16	No
*PPL2*	11	14	0.30	No	145	120	−0.27	No
*TLP21*	109	121	0.15	No	158	112	−0.49	Yes
*FKBP16‐2*	3	7	1.03	Yes	101	59	−0.78	Yes
*PsbQ‐like*	8	10	0.40	No	188	144	−0.38	Yes
*PGRL1A*	23	29	0.32	No	312	266	−0.23	No
*PGRL1B*	5	6	0.15	No	16	18	0.16	No
*PGR5*	50	56	0.15	No	190	200	0.08	No

Genes were selected by >2‐fold change with significant cutoff (yes or no) in at least one sample between 1 and 7 days. NDH‐related genes were described previously (Suorsa et al., 2009). *NDHM* to *NDH18*: genes encoding NDH subunits; *CRR1* to *PsbQ‐like*: genes encoding auxiliary proteins for the NDH complex; *PGRL1A* to *PGR5*: genes encoding PGR proteins.

a Fold change (in log2 scale).

b Significant with *q*‐value <.05.

## 4 DISCUSSION

Sigma factor protein 2 is a transcriptional regulator that regulates chloroplast gene expression and impacts retrograde signaling‐mediated regulation of nuclear‐encoded photosynthesis genes (Woodson et al., 2013). Recently, we hypothesized that SIG2 is required for phytochrome‐mediated photomorphogenesis and growth, in addition to known roles of SIG2 in plastid development (Oh & Montgomery, 2013). The contribution of SIG2 to photomorphogenesis (e.g., inhibition of hypocotyl elongation and expansion of cotyledons) and regulation of some nuclear genes (e.g., *phytochrome‐interacting factor 4*,* PIF4*;* Elongated Hypocotyl 2*, and *HY2*) appears to be R‐light‐specific (Oh & Montgomery, 2013). Compared to chloroplast genes (i.e., mostly plastid‐encoded RNA polymerase‐dependent) directly transcribed by SIG2 (Nagashima et al., 2004), we have limited knowledge about the nuclear genes targeted by SIG2‐mediated retrograde signaling which impact both photosynthesis‐ and photomorphogenesis‐dependent phenotypes in red light. Previous microarray analysis indicates that only 83 genes (0.3% out of whole genome) are misregulated in white‐light‐grown 2‐day‐old *sig2‐2* (Woodson et al., 2013). Our RNA‐Seq analysis identified a subset of nuclear genes (2% from 1 day sample, 7% from 7 days sample, out of the whole genome) that are misregulated in *sig2‐2* mutant compared to WT grown in R light (Figure 1a), suggesting a much greater impact of R‐dependent *SIG2* expression on plants than previously recognized. We compared the misregulated genes in two distinct developmental stages of seedlings, 1‐ and 7‐day‐old, and found that only 106 genes were misregulated in both stages (Figure 1a), suggesting a distinctive role for SIG2 in the transcriptional regulation of developmentally associated genes, that is, early and later in seedling development.

### 4.1 Role of SIG2 in light‐mediated GA signaling during seedling development

Functional analysis of genes misregulated in *sig2‐2* under R‐light conditions suggested a potential retrograde signaling‐dependent role of SIG2 in GA homeostasis or signaling: genes related to GA metabolic process, GA biosynthetic process, and cellular responses to GA stimulus were overrepresented in the misregulated gene sets from *sig2‐2* vs. WT (Figure 1e). In the phytochrome‐mediated light signaling pathway, SIG2 is required for the negative regulation of *PIF4*, which encodes a negative regulator of photomorphogenesis (Oh & Montgomery, 2013). The activity of PIF4 on gene transcription for photomorphogenesis depends on the abundance of PIF‐binding protein, DELLA, and the abundance of DELLA protein is increased by reduced levels of GA after light treatment in plants (Achard et al., 2007; Li et al., 2016). Thus, it is plausible that a transcriptional role of SIG2 in the regulation of GA signal‐related genes may be important for PIF‐mediated photomorphogenesis through DELLA‐associated GA signaling. *sig2‐2* and *sig2‐4* mutants exhibited reduced sensitivity to exogenous GA_3_ but similar responses to PAC, a GA biosynthesis inhibitor, compared to WT, based on a hypocotyl growth assay (Figure 2). Of note, *phyB* mutants exhibit longer hypocotyls than WT when treated with exogenous GA_3_, an inhibition of hypocotyl similar to WT when treated with PAC, and no difference in endogenous GA levels was apparent between *phyB* mutant and WT, all of which suggest a role for phytochrome in responsiveness to GA, rather than in changing total intracellular GA levels (Reed, Foster, Morgan, & Chory, 1996). In our RNA‐Seq analysis of *sig2‐2*, two genes encoding GA 3‐oxidases (*GA3OX1*,* At1 g15550*;* GA3OX2*, and *At1 g80340*), which function in converting inactive precursor GAs to their bioactive forms but which are also GA‐responsive genes, were downregulated (3.2‐fold, 2.5‐fold, respectively) in 7‐day‐old *sig2‐2* mutant (Table S5), and the downregulation of *GA3OX1* was validated by semiquantitative RT‐PCR analysis (Figure S3). Given the prior association of downregulation of *GA3OX1* in response to GA application (Thomas et al., 1999), this could indicate higher accumulation of GA in strong *sig2* mutants, which would correspond to the longer hypocotyls observed for these lines and their insensitivity to treatment relative to WT in GA‐dependent hypocotyl elongation assays. Of note, phytochromes positively regulate *GA3OX1* and *GA3OX2* genes in R light during Arabidopsis seed germination (Yamaguchi, Smith, Brown, Kamiya, & Sun, 1998), supporting the idea that SIG2 is an important component in phytochrome‐dependent GA signaling via the regulation of GA biosynthetic genes.

### 4.2 SIG2 is involved in H_2_O_2_‐mediated oxidative stress response

Our RNA‐Seq analysis identified a significant number of stress‐, ROS‐, and H_2_O_2_‐related genes that were differentially regulated in *sig2‐2* mutant compared to WT (Figures 1b and c), and in particular, the level of H_2_O_2_ was high (~3‐fold) in the *sig2‐2* and *sig2‐4* mutants at the young (7 days) or more mature (40 days) developmental stages (Figure 3). Similar to stress‐responsive SIG5, SIG2 appears to play an important role in stress signaling by transcriptional regulation of stress (H_2_O_2_)‐related genes. Plants generate H_2_O_2_ during oxygenic processes of cell metabolism or under stress conditions in most cell compartments, including chloroplasts, mitochondria, and peroxisomes, and then H_2_O_2_ can serve as a signal for cellular responses or cause oxidative damage to the cell (Halliwell, 2006). In chloroplasts, the oxygenic reactions during photosynthetic electron transfer generate superoxide radicals, which are converted to H_2_O_2_ by superoxide dismutase, whereas in peroxisomes, photorespiration‐related glycolate oxidase is involved in the generation of H_2_O_2_ (Saxena, Srikanth, & Chen, 2016). Overexpression of glycolate oxidase (35S::GO) in chloroplasts results in high accumulation of H_2_O_2_ and growth defects, including small and pale‐green rosettes and late‐flowering (Fahnenstich, Scarpeci, Valle, Flügge, & Maurino, 2008), similar to *sig2* mutants (Oh & Montgomery, 2013). Recent work has shown that mutants that display elevated H_2_O_2_ also show distinct effects on photosynthetic performance, in particular, on cyclic electron flow (CEF), which is important for balancing the ATP/NADPH energy budget of photosynthesis (Strand et al., 2015). Intriguingly, *sig2* showed a similar phenotypic pattern, with suppressed LEF and elevated CEF (Figure 5), suggesting possible regulatory or metabolic links between H_2_O_2_ and CEF. For instance, H_2_O_2_ may directly activate CEF enzymes or it may induce ATP synthesis to counteract a deficit in the production of ATP relative to NADPH (Strand, Fisher, & Kramer, 2016).

### 4.3 SIG2 contributes to the retrograde signaling‐dependent regulation of photosynthesis‐related genes in red light

Our functional classification from RNA‐Seq analysis suggested that SIG2 is required for the retrograde‐associated regulation of photosynthesis‐related genes in red light (Figures 1b and d) as expected based on prior analyses (Woodson et al., 2013). Additionally, photosystem I (PSI), PSII, and chlorophyll biosynthesis genes were misregulated in the *sig2‐2* mutant in red light, in particular (Table 1). In the light‐induced chlorophyll biosynthesis pathway, protochlorophyllide (Pchlide) is converted to a precursor of chlorophyll, chlorophyllide (Chlide) by POR (protochlorophyllide oxidoreductase), while in the dark, Pchlide along with POR enzyme is abundant (Griffiths, 1978). In the light, Pchlide can interact with oxygen, resulting in production of reactive oxygen species (ROS); thus, fine regulation of this POR gene/enzyme is crucial for survival (Reinbothe & Reinbothe, 1996). *sig2* mutants accumulate low levels of chlorophyll (Oh & Montgomery, 2013), corresponding to the downregulation of *PORA* and *PORC* genes in light‐grown 7‐day‐old seedlings (Table 1). Unlike the downregulation of *PORA* in 7‐d‐old seedlings, this gene was upregulated in 1‐day‐old seedlings, in which chlorophyll biosynthesis is likely induced by light at the very young developmental stage (Table 1). This observation is supported by the fact that PORA acts at the very early stage of dark‐to‐light transition, and the amount of PORA protein is reduced rapidly after illumination (Forreiter, van Cleve, Schmidt, & Apel, 1991).

Similar to a *phyB* mutant (Campos et al., 2016), *sig2‐2* and *sig2‐4* mutants exhibited low photosystem II efficiency (Φ_II_), and nonphotochemical quenching (NPQ) and maximal quantum yield (Fv/Fm) were also disrupted under various light regimes (Figure 4b; Figure 5a–c). It was not unexpected to observe significant defects in photosynthetic performance in *sig2* mutants because of small and chlorotic mutant phenotypes (Figure 4a); however, it was not still clear how SIG2 affects distinct aspects of photosynthesis. The accumulation of H_2_O_2_ may arise from defects in metabolism in strong *sig2* mutants that could lead to disruptions in photosynthesis that result in the accumulation of highly reactive electron transfer intermediates that produce H_2_O_2_. Alternatively, the high level of H_2_O_2_ in *sig2* mutants may cause a defect in photosynthetic performance, resulting in a change in the demands for ATP relative to NADPH, which in turn may lead to increased CEF through the NADH dehydrogenase‐like complex (NDH) (Figures 3 and 5h). Strand et al. (2015) provided evidence that H_2_O_2_, a product of imbalanced redox reactions in chloroplasts, can activate NDH‐mediated CEF in vivo, and in a mutant deficient of the NDH complex, the level of CEF is not increased after H_2_O_2_ treatment. The high level of H_2_O_2_ with elevated levels of CEF has also been observed in glycolate oxidase overexpression plants and in an *hcef1* mutant (*high cyclic electron flow 1*), and a high level of NDH protein content in the glycolate oxidase overexpression plants also was observed (Livingston et al., 2010; Strand et al., 2015). Analysis of an additional high cyclic mutant, that is, *hcef2*, indicated that disruptions in the expression of chloroplast proteins due to a mutation of a gene coding for a plastid targeted tRNA‐editing enzyme resulted in elevated H_2_O_2_ and CEF (Strand et al., 2017). Notably, *hcef2* did not show a reduction in the accumulation of NDH protein (Strand et al., 2017). In our studies, we observed reduced accumulation of NDH 18 protein in *sig2‐2* and *sig2‐4* mutants (Figure 6) that corresponds to a moderate downregulation (1.6‐fold) of the *NDH18* gene in 7‐day‐old *sig2‐2* mutant seedlings (Table 3). NDH is a supercomplex, consisting of proteins, encoded in both nucleus and plastid genomes, such as photosystems. With either overexpression or underexpression of a component of a multisubunit complex such as NDH or the photosystems, an imbalance in the stoichiometry of components could result in complexes with decreased or modified functions. In this regard, NDH18 is plant‐specific protein that has been shown specifically to be important for NDH complex stability (Peng et al., 2009). Thus, the reduced amount of NDH18 protein in *sig2* mutants likely causes a defect of NDH‐mediated CEF. Furthermore, the relative content of NDH versus LEF‐specific components, especially PSII, may be critical.

In *sig2‐2* and *sig2‐4* mutants, linear electron flow (LEF) was low (Figure 5d) and CEF was high (Figure 5h), similar to glycolate oxidase overexpression plants (Strand et al., 2015). LEF is required for net reduction in NADPH and also generates thylakoid proton motive force (*pmf*) that drives the synthesis of ATP; both NADPH and ATP are required to power the Calvin–Benson–Bassham (CBB) cycle. LEF‐generated ATP is also involved in activating NPQ (i.e., qE), a photoprotective mechanism, to dissipate stressful excessive light energy during photosynthesis (Eberhard, Finazzi, & Wollman, 2008). The amount of ATP generated by LEF alone is not sufficient for the proper ratio with NADPH for photosynthesis; thus, additional ATP‐generating mechanisms (e.g., CEF) are necessary (Kramer & Evans, 2011). As the ratio of ATP and NADPH can vary based on environmental conditions or developmental state, synthesis of ATP by CEF could also be different in various environmental conditions or even in different species (Kramer & Evans, 2011). For example, drought stress can cause high levels of CEF, along with high production of H_2_O_2_ (Cruz de Carvalho, 2008; Kohzuma et al., 2009). We hypothesize that SIG2 is a light stress‐responsible component during retrograde signaling through the transcriptional regulation of H_2_O_2_ and photosynthesis (i.e., CEF)‐related genes. Tests of the physiological responses upon environmental stresses (e.g., high light, drought, and salt.) in *sig2* mutants and genetic analysis of SIG2 and known CEF components (e.g., glycolate oxidase and NDH) would provide additional details for the role of SIG2 on H_2_O_2_‐mediated CEF phenomenon during photosynthesis.

Using RNA‐Seq analysis and physiological approaches, we discovered the importance of SIG2 in red‐light‐dependent photomorphogenic development and photosynthesis. As a retrograde signaling component, SIG2 regulated a subset of nuclear genes, including hormonal genes, stress‐responsive genes, and photosynthesis‐related genes under these conditions. Indeed, *sig2* mutants exhibited reduced sensitivity to GA, high levels of H_2_O_2_, and defects in photosynthetic performance, including loss of overall photosynthetic capacity accompanied by elevated CEF. SIG2, thus, contributes to growth and development by regulating hormonal responses that are critical for the optimal development and function of the photosynthetic apparatus under red‐light conditions associated specifically with phytochrome‐dependent regulation of photomorphogenesis.

## AUTHOR CONTRIBUTIONS

S.O and B.L.M designed the research; S.O. and D.S. performed research; S.O., D.S., D.M.K., J.C., and B.L.M. analyzed data; S.O. and B.L.M wrote the manuscript; and S.O., D.S., D.M.K., and B.L.M critically edited the manuscript.

## ACCESSION NUMBERS

RNA expression data are available in the NCBI Gene Expression Omnibus database under accession number GSE104518.
