# Supplementary material for: Transcriptome and phenotyping analyses support a role for chloroplast sigma factor 2 in red‐light‐dependent regulation of growth, stress, and photosynthesis
Source: Plant Direct. 2018 Feb 19;2(2):e00043. doi: 10.1002/pld3.43 (PMC6508532; doi:10.1002/pld3.43)
Supplement: Supplementary file 1 [file PLD3-2-e00043-s001.docx]

**SUPPORTING INFORMATION**

**Transcriptome and phenotyping analyses support a role for Chloroplast Sigma Factor 2 in red light-dependent regulation of growth, stress, and photosynthesis**

Authors: Sookyung Oh^1^, Deserah Strand^1†^, David M. Kramer^1,2^, Jin Chen^3^, Beronda L. Montgomery^1,2,4*^

^1^Department of Energy – Plant Research Laboratory, Michigan State University, East Lansing, MI 48824, USA

^2^Department of Biochemistry and Molecular Biology, Michigan State University, East Lansing, MI 48824, USA

^3^University of Kentucky College of Medicine, UK Medical Center MN 150, Lexington, KY40536, USA

^4^Department of Microbiology & Molecular Genetics, Michigan State University, East Lansing, MI 48824, USA.

*For correspondence: E-mail: [montg133@msu.edu](mailto:montg133@msu.edu)

†Present address: Max-Planck-Institut für Molekulare Pflanzenphysiologie, Am Mühlenberg 1, 14476 Potsdam-Golm, Germany

**Supplemental Figure 1.** Expression of eight genes encoding extensins of group IIb in *sig2-2* mutant. Value of expression levels (RPKM, Reads Per Kilobase per Million mapped reads) from RNA-Seq analysis comparing Col-0 wild-type and *sig2-2* mutant grown on MS medium under Rc (50 µmol m^-2^ s^-1^) for one day (1d) or seven days (7d) is shown. Eight extensins (EXT6-EXT13) have been classified as group IIb in Cannon et al. (2008).

**
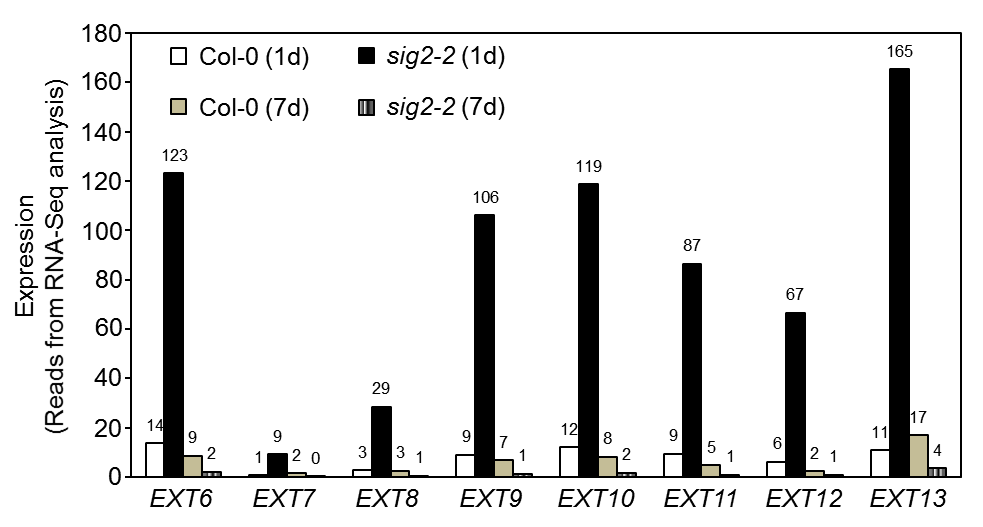
**

**Supplemental Figure 2**. Heat map analysis of Arabidopsis extension genes. The expression of Extensin (*EXT*) genes in various Arabidopsis tissues (A) or different light conditions (B) is shown. Mean-normalized values from AtGenExpress expression library (www.weigelworld.org) and BAR Heatmapper *Plus* (http://bar.utoronto.ca) were utilized for the construction of the heat map. In the different light experiments, 4-d-old seedlings were grown on MS medium in different light conditions for either 45 or 240 minutes and aerial parts of plants (i.e., cotyledons and hypocotyl) were used. 20 Arabidopsis extensin proteins have been classified into four groups, i.e., I, IIa, IIb, and IIc, in Cannon et al. (2008). Color scale represents log2 expression values, red indicates high expression; yellow, low expression.


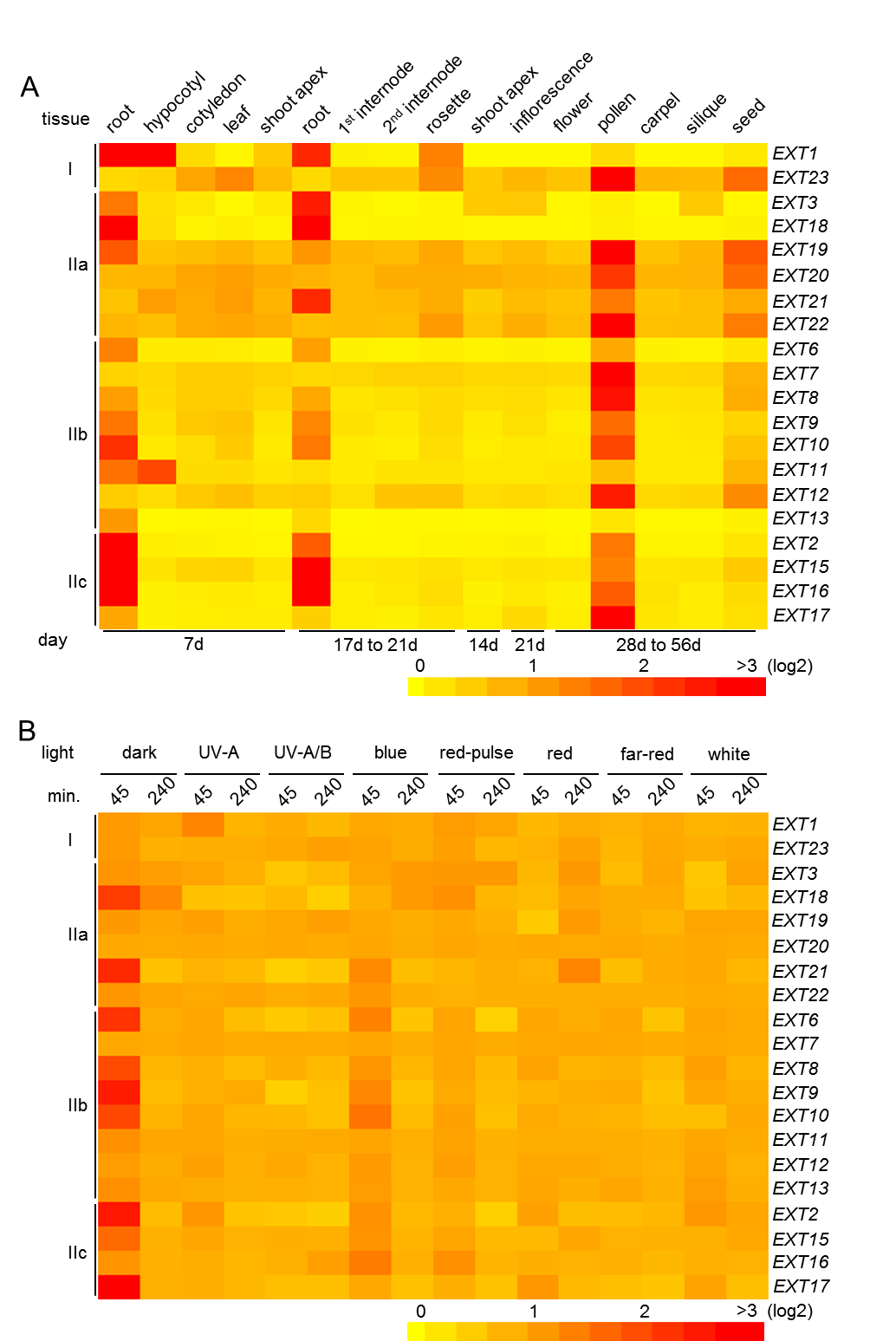


**Supplemental Figure 3**. Semi-Quantitative RT-PCR Analysis for Gibberellin 3-beta-dioxygenase 1 (*GA3OX1*, *At1g15550*) in seven-day-old Col-0 wild-type (WT) and *sig2* mutant seedlings grown on MS medium containing 1 % Suc and 0.7 % Phytoblend agar at 22 ºC under Rc (50 µmol m^-2^ s^-1^). *UBC21* gene was used as an internal control. Results are shown as three independent biological replicates.

**
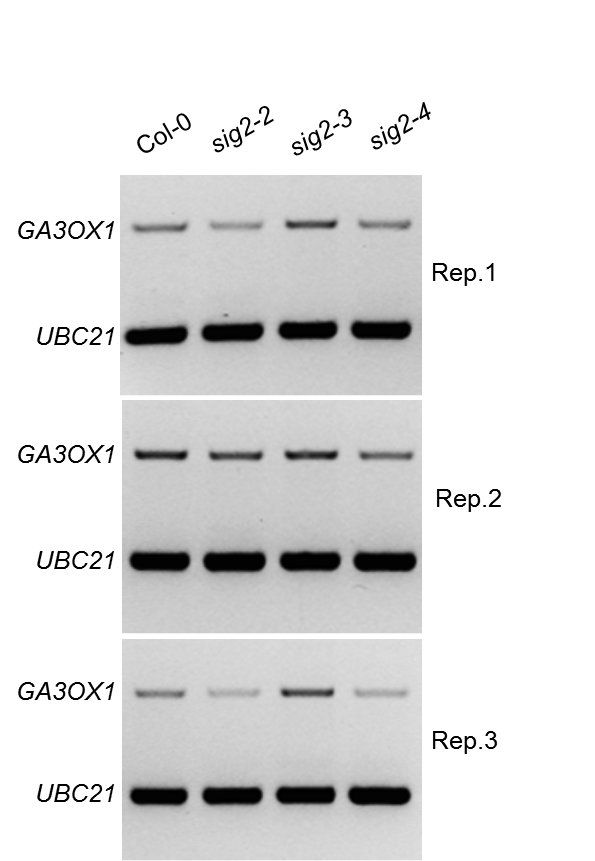
**

**Supplemental Figure 4**. Validation of RNA-Seq Data for select photosynthetic genes. Genes identified by RNA-Seq analysis were validated by quantitative RT-PCR analysis using Col-0 wild-type (WT) and *sig2-2* grown on MS medium under Rc (50 µmol m^-2^ s^-1^) for one day (1 d) or seven days (7 d). (Inset) Smaller view of the graph for 1 d. Average relative expression of *LHCB2.4* (Top panel) or *PsaE* (Bottom panel) compared to *UBC21* that was used as an internal control were shown (±SD, n=3 biological repeats).

**
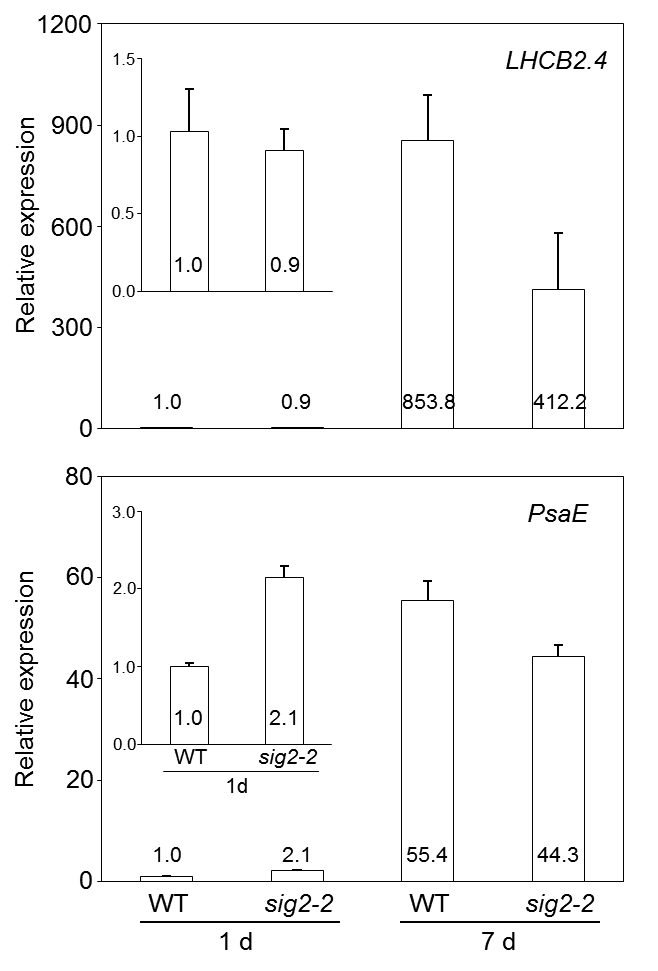
**

**Supplemental Table 1.** Primer Sequences for Quantitative RT-PCR

| **AGI number** | **Annotation** | **Primers (Forward / Reverse)** |
| --- | --- | --- |
| *At3g27690* | *LHCB2.4* | cgtgagctcgaagtgatcca/cagggaaggtgcagcctaaa |
| *At2g20260* | *PsaE* | cgtcagcagctaccggattta/ccgctctgactactagccttg |
| *At1g15550* | *GA3OX1* | ggggtgccttccaaatctcaaac/ggtgcaacacgcttttaaacaatcc |
| *At5g25760* | *UBC21* | caaatggaccgctcttatcaaag/ctgaaaaacaccgccttcgt |

| **Supplemental Table 2.** Categorization of Genes Misregulated in One-Day-Old (1 d) *sig2-2* Mutant | |  |  |
| --- | --- | --- | --- |
|  |  |  |  |
| **Category** | **Fold-change (*sig2-2*/WT vs. Genome)** | **P values** | |
| gibberellin biosynthetic process | 9.8 | 2.5E-04 | |
| diterpenoid biosynthetic process | 9.6 | 2.9E-04 | |
| trichoblast differentiation | 8.9 | 5.1E-32 | |
| response to nitrate | 8.9 | 7.1E-17 | |
| iron ion transport | 8.8 | 2.2E-09 | |
| coumarin biosynthetic process | 8.7 | 2.9E-03 | |
| cellular response to iron ion starvation | 8.6 | 1.1E-08 | |
| coumarin metabolic process | 8.6 | 3.3E-03 | |
| gibberellin metabolic process | 8.5 | 8.4E-04 | |
| diterpenoid metabolic process | 8.4 | 9.7E-04 | |
| root epidermal cell differentiation | 8.4 | 1.6E-30 | |
| nitrate transport | 8.2 | 2.3E-15 | |
| root morphogenesis | 6.9 | 2.0E-27 | |
| inorganic anion transport | 6.9 | 2.9E-15 | |
| cellular response to gibberellin stimulus | 6.8 | 1.9E-03 | |
| positive regulation of flavonoid biosynthetic process | 6.4 | 1.0E-03 | |
| root hair cell development | 6.4 | 5.8E-09 | |
| root hair elongation | 6.3 | 7.3E-08 | |
| seed dormancy process | 6.3 | 1.9E-06 | |
| dormancy process | 6.3 | 1.9E-06 | |
| trichoblast maturation | 6.2 | 2.4E-14 | |
| root hair cell differentiation | 6.2 | 2.4E-14 | |
| cellular cation homeostasis | 6.2 | 7.7E-07 | |
| cell maturation | 6.2 | 2.6E-14 | |
| epidermal cell differentiation | 6.2 | 8.8E-24 | |
| epithelial cell differentiation | 6.2 | 8.8E-24 | |
| epithelium development | 6.2 | 8.8E-24 | |
| cellular ion homeostasis | 6.1 | 3.6E-07 | |
| response to red light | 6.1 | 1.8E-03 | |
| developmental maturation | 5.9 | 9.6E-14 | |
| divalent metal ion transport | 5.9 | 2.0E-07 | |
| divalent inorganic cation transport | 5.9 | 2.4E-07 | |
| lipid storage | 5.8 | 7.5E-03 | |
| transition metal ion transport | 5.8 | 1.3E-08 | |
| cellular chemical homeostasis | 5.7 | 9.7E-07 | |
| seed maturation | 5.6 | 3.5E-06 | |
| cellular homeostasis | 5.3 | 3.9E-06 | |
| root development | 5.3 | 3.4E-22 | |
| root system development | 5.3 | 3.7E-22 | |
| regulation of flavonoid biosynthetic process | 5.2 | 7.7E-03 | |
| phenylpropanoid biosynthetic process | 5.1 | 4.1E-03 | |
| cation homeostasis | 4.9 | 2.9E-05 | |
| epidermis development | 4.9 | 2.4E-21 | |
| skin development | 4.9 | 2.4E-21 | |
| cysteine biosynthetic process | 4.9 | 8.0E-05 | |
| cysteine metabolic process | 4.8 | 9.2E-05 | |
| serine family amino acid biosynthetic process | 4.8 | 1.1E-04 | |
| ion homeostasis | 4.6 | 3.9E-05 | |
| metal ion transport | 4.6 | 2.9E-13 | |
| phenylpropanoid metabolic process | 4.6 | 2.5E-03 | |
| hemicellulose metabolic process | 4.1 | 8.3E-03 | |
| seed germination | 4.1 | 2.1E-03 | |
| Photosynthesis | 4.1 | 1.9E-07 | |
| regulation of reactive oxygen species metabolic process | 4.0 | 9.8E-03 | |
| anion transport | 4.0 | 1.1E-10 | |
| lipid localization | 4.0 | 1.5E-03 | |
| photosynthesis, light reaction | 3.9 | 1.2E-04 | |
| serine family amino acid metabolic process | 3.9 | 1.9E-03 | |
| seedling development | 3.9 | 3.8E-03 | |
| chemical homeostasis | 3.8 | 5.9E-04 | |
| secondary metabolite biosynthetic process | 3.8 | 1.6E-04 | |
| cation transport | 3.8 | 7.2E-13 | |
| terpenoid metabolic process | 3.7 | 2.4E-03 | |
| cell wall macromolecule metabolic process | 3.6 | 1.5E-03 | |
| developmental cell growth | 3.6 | 1.1E-05 | |
| ion transport | 3.5 | 3.1E-16 | |
| tissue development | 3.4 | 5.0E-17 | |
| cell development | 3.4 | 6.2E-09 | |
| nitrogen compound transport | 3.4 | 1.2E-09 | |
| response to nitrogen compound | 3.3 | 1.7E-09 | |
| cellular response to starvation | 3.3 | 6.1E-03 | |
| response to starvation | 3.2 | 8.4E-03 | |
| cell differentiation | 3.1 | 9.1E-13 | |
| developmental growth involved in morphogenesis | 3.1 | 2.2E-05 | |
| response to inorganic substance | 2.9 | 3.2E-12 | |
| cell growth | 2.8 | 4.9E-05 | |
| secondary metabolic process | 2.8 | 5.5E-04 | |
| developmental growth | 2.7 | 5.6E-05 | |
| anatomical structure morphogenesis | 2.7 | 7.7E-14 | |
| cell wall organization or biogenesis | 2.7 | 2.1E-05 | |
| cellular developmental process | 2.6 | 2.6E-10 | |
| response to oxygen-containing compound | 2.6 | 2.5E-18 | |
| response to cold | 2.6 | 4.0E-03 | |
| organic acid biosynthetic process | 2.6 | 2.6E-06 | |
| carboxylic acid biosynthetic process | 2.6 | 2.6E-06 | |
| generation of precursor metabolites and energy | 2.6 | 7.0E-03 | |
| cellular response to hormone stimulus | 2.5 | 9.1E-04 | |
| cellular response to endogenous stimulus | 2.5 | 1.0E-03 | |
| cellular response to oxygen-containing compound | 2.4 | 2.3E-04 | |
| regulation of biological quality | 2.4 | 2.0E-05 | |
| Growth | 2.4 | 2.6E-04 | |
| response to temperature stimulus | 2.4 | 5.5E-04 | |
| organ development | 2.4 | 1.6E-10 | |
| small molecule biosynthetic process | 2.3 | 4.8E-06 | |
| response to osmotic stress | 2.3 | 6.6E-03 | |
| response to lipid | 2.3 | 5.6E-03 | |
| cellular response to organic substance | 2.3 | 6.5E-05 | |
| response to hormone | 2.2 | 4.9E-06 | |
| response to endogenous stimulus | 2.2 | 4.7E-07 | |
| single-organism transport | 2.2 | 5.5E-11 | |
| response to chemical | 2.2 | 1.0E-16 | |
| Localization | 2.1 | 7.6E-13 | |
| cellular response to chemical stimulus | 2.1 | 2.8E-04 | |
| establishment of localization | 2.1 | 2.1E-10 | |
| system development | 2.0 | 3.8E-10 | |
| Transport | 2.0 | 1.4E-09 | |
| response to organic substance | 2.0 | 4.5E-08 | |
| defense response | 2.0 | 8.4E-04 | |
| cell communication | 1.9 | 1.2E-04 | |
| multicellular organismal development | 1.9 | 1.1E-09 | |
| response to stress | 1.9 | 1.4E-09 | |
| carboxylic acid metabolic process | 1.9 | 6.0E-03 | |
| anatomical structure development | 1.8 | 3.3E-09 | |
| single-multicellular organism process | 1.8 | 4.3E-09 | |
| oxoacid metabolic process | 1.8 | 7.6E-03 | |
| organic acid metabolic process | 1.8 | 7.7E-03 | |
| developmental process | 1.8 | 3.0E-10 | |
| single-organism developmental process | 1.8 | 1.1E-09 | |
| cellular response to stimulus | 1.8 | 2.5E-05 | |
| multicellular organismal process | 1.8 | 2.2E-08 | |
| response to abiotic stimulus | 1.7 | 5.4E-04 | |
| single-organism biosynthetic process | 1.7 | 8.5E-04 | |
| response to stimulus | 1.7 | 5.1E-11 | |
| single-organism cellular process | 1.6 | 7.9E-14 | |
| single-organism process | 1.6 | 1.3E-17 | |
| biological regulation | 1.4 | 5.4E-03 | |
| cellular process | 1.3 | 2.5E-04 | |
| **Supplemental Table 3.** Categorization of Genes Misregulated in Seven-Day-Old (7 d) *sig2-2* Mutant | |  |  |
|  |  |  |  |
|  |  |  |  |
|  |  |  |  |
| **Category** | **Fold-change (*sig2-2*/WT vs. Genome)** | **P values** | |
| spindle assembly | 6.9 | 2.8E-08 | |
| spindle organization | 6.5 | 9.6E-08 | |
| regulation of G2/M transition of mitotic cell cycle | 6.4 | 3.7E-10 | |
| histone phosphorylation | 6.3 | 1.7E-09 | |
| G2/M transition of mitotic cell cycle | 6.3 | 5.5E-10 | |
| sepal morphogenesis | 5.9 | 7.6E-10 | |
| sepal formation | 5.9 | 7.6E-10 | |
| regulation of cell cycle phase transition | 5.9 | 7.6E-10 | |
| regulation of mitotic cell cycle phase transition | 5.9 | 7.6E-10 | |
| organelle assembly | 5.9 | 6.1E-07 | |
| cell cycle phase transition | 5.8 | 3.5E-10 | |
| mitotic cell cycle phase transition | 5.8 | 3.5E-10 | |
| petal formation | 5.8 | 1.1E-09 | |
| iron ion homeostasis | 5.5 | 9.5E-03 | |
| sepal development | 5.5 | 5.5E-09 | |
| flower calyx development | 5.5 | 5.5E-09 | |
| anthocyanin-containing compound biosynthetic process | 5.5 | 3.4E-07 | |
| response to absence of light | 5.3 | 5.7E-03 | |
| petal morphogenesis | 5.0 | 1.4E-08 | |
| mitotic cytokinesis | 5.0 | 5.6E-22 | |
| cytokinesis by cell plate formation | 5.0 | 5.6E-22 | |
| cytokinetic process | 5.0 | 5.6E-22 | |
| mitotic cytokinetic process | 5.0 | 5.6E-22 | |
| cytokinesis | 4.6 | 1.1E-20 | |
| response to sucrose | 4.6 | 9.0E-19 | |
| response to disaccharide | 4.6 | 9.0E-19 | |
| response to fructose | 4.3 | 1.8E-10 | |
| regulation of mitotic cell cycle | 4.3 | 5.8E-07 | |
| indoleacetic acid biosynthetic process | 4.2 | 3.9E-07 | |
| response to insect | 4.2 | 1.1E-04 | |
| indoleacetic acid metabolic process | 4.2 | 4.9E-07 | |
| S-glycoside biosynthetic process | 4.2 | 5.9E-12 | |
| glycosinolate biosynthetic process | 4.2 | 5.9E-12 | |
| glucosinolate biosynthetic process | 4.2 | 5.9E-12 | |
| response to UV-B | 4.1 | 4.8E-06 | |
| response to monosaccharide | 4.0 | 3.8E-11 | |
| microtubule cytoskeleton organization | 4.0 | 2.8E-15 | |
| regulation of DNA replication | 4.0 | 5.0E-08 | |
| response to hexose | 3.9 | 4.3E-10 | |
| anthocyanin-containing compound metabolic process | 3.9 | 1.9E-04 | |
| response to karrikin | 3.9 | 7.1E-07 | |
| auxin biosynthetic process | 3.9 | 1.6E-06 | |
| transition metal ion homeostasis | 3.9 | 8.1E-03 | |
| S-glycoside metabolic process | 3.8 | 6.5E-12 | |
| glycosinolate metabolic process | 3.8 | 6.5E-12 | |
| glucosinolate metabolic process | 3.8 | 6.5E-12 | |
| response to cyclopentenone | 3.8 | 2.3E-06 | |
| microtubule-based process | 3.8 | 5.3E-15 | |
| petal development | 3.8 | 9.9E-06 | |
| corolla development | 3.8 | 9.9E-06 | |
| respiratory burst involved in defense response | 3.8 | 5.3E-06 | |
| respiratory burst | 3.8 | 5.3E-06 | |
| indole-containing compound biosynthetic process | 3.7 | 2.1E-06 | |
| histone H3-K9 methylation | 3.7 | 3.3E-09 | |
| indole-containing compound metabolic process | 3.6 | 1.2E-06 | |
| auxin metabolic process | 3.5 | 1.2E-05 | |
| glycosyl compound biosynthetic process | 3.4 | 5.2E-09 | |
| regulation of cell cycle process | 3.4 | 4.1E-05 | |
| organ senescence | 3.3 | 4.8E-03 | |
| response to nitrate | 3.3 | 1.9E-07 | |
| nitrate transport | 3.3 | 1.6E-07 | |
| cellular response to iron ion starvation | 3.2 | 2.8E-03 | |
| secondary metabolite biosynthetic process | 3.2 | 5.0E-12 | |
| histone lysine methylation | 3.1 | 1.5E-07 | |
| amino acid transport | 3.1 | 1.2E-08 | |
| inorganic anion transport | 3.1 | 2.8E-09 | |
| floral organ formation | 3.1 | 6.2E-05 | |
| response to carbohydrate | 3.0 | 1.0E-13 | |
| organic acid transport | 3.0 | 5.4E-09 | |
| carboxylic acid transport | 3.0 | 5.4E-09 | |
| response to chitin | 3.0 | 4.9E-14 | |
| response to wounding | 3.0 | 5.9E-11 | |
| response to organonitrogen compound | 3.0 | 3.1E-14 | |
| organic anion transport | 3.0 | 3.4E-09 | |
| cell division | 3.0 | 1.6E-12 | |
| regulation of DNA metabolic process | 2.9 | 2.2E-05 | |
| flavonoid biosynthetic process | 2.9 | 1.2E-05 | |
| cellular hormone metabolic process | 2.9 | 6.7E-04 | |
| cellular amino acid catabolic process | 2.9 | 1.1E-03 | |
| alpha-amino acid catabolic process | 2.8 | 2.0E-03 | |
| mitotic cell cycle | 2.8 | 9.1E-12 | |
| toxin catabolic process | 2.8 | 5.2E-05 | |
| secondary metabolite catabolic process | 2.8 | 5.2E-05 | |
| secondary metabolic process | 2.8 | 1.7E-18 | |
| aging | 2.8 | 3.7E-03 | |
| toxin metabolic process | 2.8 | 7.3E-05 | |
| response to water deprivation | 2.7 | 2.2E-10 | |
| negative regulation of programmed cell death | 2.7 | 4.5E-03 | |
| anion transport | 2.7 | 6.9E-15 | |
| cellular response to extracellular stimulus | 2.7 | 3.1E-09 | |
| cellular response to starvation | 2.7 | 9.5E-08 | |
| cellular response to external stimulus | 2.7 | 3.5E-09 | |
| cellular response to nutrient levels | 2.7 | 4.5E-08 | |
| response to water | 2.7 | 4.8E-10 | |
| response to extracellular stimulus | 2.7 | 2.3E-09 | |
| response to nutrient levels | 2.7 | 3.0E-08 | |
| negative regulation of cell death | 2.7 | 7.0E-03 | |
| cell proliferation | 2.7 | 2.4E-05 | |
| response to starvation | 2.6 | 2.2E-07 | |
| regulation of hydrogen peroxide metabolic process | 2.6 | 3.7E-03 | |
| flavonoid metabolic process | 2.6 | 1.1E-04 | |
| response to salicylic acid | 2.6 | 1.0E-10 | |
| chromatin silencing | 2.6 | 1.8E-04 | |
| defense response to fungus | 2.6 | 9.4E-08 | |
| glycosyl compound metabolic process | 2.6 | 3.7E-06 | |
| response to nitrogen compound | 2.6 | 1.2E-17 | |
| negative regulation of gene expression, epigenetic | 2.6 | 3.1E-04 | |
| response to jasmonic acid | 2.5 | 1.6E-09 | |
| histone methylation | 2.5 | 7.1E-05 | |
| floral organ morphogenesis | 2.5 | 1.4E-03 | |
| post-embryonic organ morphogenesis | 2.5 | 1.4E-03 | |
| regulation of reactive oxygen species metabolic process | 2.5 | 6.4E-03 | |
| protein methylation | 2.5 | 9.5E-05 | |
| protein alkylation | 2.5 | 9.5E-05 | |
| histone modification | 2.5 | 3.6E-06 | |
| organ formation | 2.5 | 1.9E-03 | |
| regulation of cell cycle | 2.5 | 1.0E-04 | |
| response to UV | 2.4 | 2.5E-03 | |
| response to ethylene | 2.4 | 1.6E-05 | |
| DNA replication | 2.4 | 1.3E-04 | |
| defense response to bacterium | 2.4 | 1.7E-06 | |
| regulation of cellular response to stress | 2.4 | 9.0E-06 | |
| negative regulation of transcription, DNA-templated | 2.4 | 8.1E-06 | |
| negative regulation of RNA metabolic process | 2.4 | 8.1E-06 | |
| cytoskeleton organization | 2.4 | 1.9E-07 | |
| systemic acquired resistance, salicylic acid mediated signaling pathway | 2.4 | 3.5E-03 | |
| response to oxygen-containing compound | 2.3 | 1.8E-46 | |
| regulation of plant-type hypersensitive response | 2.3 | 5.0E-05 | |
| monocarboxylic acid biosynthetic process | 2.3 | 1.0E-08 | |
| cell cycle process | 2.3 | 5.8E-09 | |
| response to fungus | 2.3 | 3.4E-08 | |
| cellular response to salicylic acid stimulus | 2.3 | 9.4E-05 | |
| protein targeting to membrane | 2.3 | 7.1E-05 | |
| protein localization to membrane | 2.3 | 8.4E-05 | |
| establishment of protein localization to membrane | 2.3 | 8.4E-05 | |
| negative regulation of macromolecule biosynthetic process | 2.3 | 2.7E-05 | |
| negative regulation of nucleobase-containing compound metabolic process | 2.3 | 2.7E-05 | |
| negative regulation of cellular macromolecule biosynthetic process | 2.3 | 2.7E-05 | |
| covalent chromatin modification | 2.3 | 4.2E-05 | |
| plant-type hypersensitive response | 2.3 | 4.5E-05 | |
| regulation of programmed cell death | 2.3 | 6.5E-05 | |
| regulation of cell death | 2.3 | 4.9E-05 | |
| host programmed cell death induced by symbiont | 2.3 | 4.9E-05 | |
| negative regulation of nitrogen compound metabolic process | 2.3 | 3.7E-05 | |
| regulation of innate immune response | 2.3 | 3.3E-05 | |
| response to organic cyclic compound | 2.2 | 2.6E-12 | |
| regulation of immune response | 2.2 | 3.8E-05 | |
| response to bacterium | 2.2 | 4.7E-08 | |
| negative regulation of cellular biosynthetic process | 2.2 | 6.0E-05 | |
| regulation of immune system process | 2.2 | 4.8E-05 | |
| salicylic acid mediated signaling pathway | 2.2 | 5.2E-04 | |
| negative regulation of biosynthetic process | 2.2 | 8.8E-05 | |
| cell death | 2.2 | 5.8E-06 | |
| death | 2.2 | 5.8E-06 | |
| programmed cell death | 2.2 | 3.3E-05 | |
| anatomical structure formation involved in morphogenesis | 2.2 | 2.3E-03 | |
| organonitrogen compound catabolic process | 2.2 | 4.7E-03 | |
| nitrogen compound transport | 2.2 | 2.3E-09 | |
| chromatin modification | 2.1 | 3.9E-04 | |
| regulation of response to stress | 2.1 | 1.1E-05 | |
| single-organism localization | 2.1 | 1.8E-04 | |
| single-organism cellular localization | 2.1 | 1.8E-04 | |
| regulation of defense response | 2.1 | 4.8E-05 | |
| systemic acquired resistance | 2.0 | 1.0E-03 | |
| response to oxidative stress | 2.0 | 8.3E-05 | |
| response to organic substance | 2.0 | 8.1E-33 | |
| negative regulation of cellular metabolic process | 2.0 | 1.1E-03 | |
| ion transport | 2.0 | 6.6E-11 | |
| response to inorganic substance | 2.0 | 1.6E-13 | |
| response to endogenous stimulus | 2.0 | 1.2E-18 | |
| negative regulation of cellular process | 2.0 | 1.6E-06 | |
| sulfur compound biosynthetic process | 1.9 | 5.6E-03 | |
| cell cycle | 1.9 | 2.6E-06 | |
| response to external biotic stimulus | 1.9 | 2.0E-13 | |
| response to other organism | 1.9 | 2.0E-13 | |
| response to biotic stimulus | 1.9 | 2.1E-13 | |
| immune system process | 1.9 | 3.1E-08 | |
| regulation of response to stimulus | 1.9 | 2.7E-05 | |
| cellular response to hormone stimulus | 1.9 | 4.1E-06 | |
| defense response to other organism | 1.9 | 1.5E-08 | |
| chromatin organization | 1.9 | 6.5E-03 | |
| sulfur compound metabolic process | 1.9 | 2.0E-03 | |
| cellular response to endogenous stimulus | 1.9 | 5.4E-06 | |
| response to chemical | 1.9 | 1.6E-36 | |
| defense response | 1.9 | 2.4E-13 | |
| response to external stimulus | 1.9 | 2.1E-18 | |
| hormone-mediated signaling pathway | 1.9 | 3.6E-05 | |
| cellular response to oxygen-containing compound | 1.8 | 2.8E-06 | |
| response to abscisic acid | 1.8 | 2.5E-03 | |
| cellular response to stress | 1.8 | 2.1E-10 | |
| defense response, incompatible interaction | 1.8 | 6.5E-03 | |
| response to hormone | 1.8 | 7.2E-12 | |
| innate immune response | 1.8 | 2.0E-05 | |
| response to alcohol | 1.8 | 7.1E-04 | |
| immune response | 1.8 | 2.7E-05 | |
| monocarboxylic acid metabolic process | 1.8 | 6.0E-07 | |
| response to osmotic stress | 1.8 | 5.9E-04 | |
| cellular response to chemical stimulus | 1.8 | 4.6E-08 | |
| response to salt stress | 1.8 | 2.0E-03 | |
| response to abiotic stimulus | 1.7 | 1.1E-17 | |
| phosphorylation | 1.7 | 6.4E-03 | |
| negative regulation of biological process | 1.7 | 1.6E-06 | |
| response to lipid | 1.7 | 1.4E-03 | |
| response to stress | 1.7 | 1.4E-25 | |
| cellular response to organic substance | 1.7 | 5.4E-06 | |
| cation transport | 1.7 | 5.9E-03 | |
| organic acid biosynthetic process | 1.7 | 1.7E-04 | |
| carboxylic acid biosynthetic process | 1.7 | 1.7E-04 | |
| cell communication | 1.7 | 9.7E-10 | |
| small molecule biosynthetic process | 1.7 | 1.1E-05 | |
| response to stimulus | 1.6 | 1.4E-38 | |
| response to light stimulus | 1.6 | 6.7E-04 | |
| oxoacid metabolic process | 1.6 | 3.7E-06 | |
| organic acid metabolic process | 1.6 | 3.9E-06 | |
| response to radiation | 1.6 | 8.7E-04 | |
| multi-organism process | 1.6 | 7.8E-07 | |
| regulation of transcription, DNA-templated | 1.6 | 4.3E-06 | |
| regulation of RNA biosynthetic process | 1.6 | 4.3E-06 | |
| single-organism catabolic process | 1.6 | 3.4E-03 | |
| cellular response to stimulus | 1.6 | 1.7E-09 | |
| regulation of RNA metabolic process | 1.5 | 6.2E-06 | |
| regulation of nucleobase-containing compound metabolic process | 1.5 | 2.3E-06 | |
| carboxylic acid metabolic process | 1.5 | 5.5E-05 | |
| regulation of cellular metabolic process | 1.5 | 2.0E-07 | |
| regulation of nitrogen compound metabolic process | 1.5 | 4.6E-06 | |
| regulation of macromolecule biosynthetic process | 1.5 | 7.9E-06 | |
| regulation of cellular macromolecule biosynthetic process | 1.5 | 7.9E-06 | |
| regulation of cellular biosynthetic process | 1.5 | 5.7E-06 | |
| signal transduction | 1.5 | 6.3E-04 | |
| regulation of biosynthetic process | 1.5 | 4.8E-06 | |
| organ development | 1.5 | 6.8E-05 | |
| single organism signaling | 1.5 | 5.5E-03 | |
| signaling | 1.5 | 5.7E-03 | |
| regulation of primary metabolic process | 1.5 | 6.5E-05 | |
| regulation of gene expression | 1.4 | 2.8E-04 | |
| single-organism biosynthetic process | 1.4 | 9.3E-07 | |
| regulation of cellular process | 1.4 | 1.5E-09 | |
| single-organism metabolic process | 1.4 | 1.3E-13 | |
| organic cyclic compound biosynthetic process | 1.4 | 7.9E-07 | |
| single-organism transport | 1.4 | 3.8E-05 | |
| regulation of metabolic process | 1.4 | 1.1E-05 | |
| transcription, DNA-templated | 1.4 | 1.1E-03 | |
| aromatic compound biosynthetic process | 1.4 | 5.5E-06 | |
| RNA biosynthetic process | 1.4 | 1.1E-03 | |
| biological regulation | 1.4 | 5.6E-13 | |
| single-organism cellular process | 1.4 | 4.5E-21 | |
| regulation of macromolecule metabolic process | 1.4 | 9.1E-04 | |
| cellular nitrogen compound biosynthetic process | 1.4 | 2.1E-04 | |
| regulation of biological process | 1.4 | 1.3E-09 | |
| heterocycle biosynthetic process | 1.4 | 4.2E-04 | |
| small molecule metabolic process | 1.4 | 9.8E-03 | |
| transport | 1.3 | 2.1E-03 | |
| single-organism process | 1.3 | 1.7E-23 | |
| establishment of localization | 1.3 | 6.0E-03 | |
| cellular process | 1.1 | 5.4E-04 | |

**Supplemental Table 4.** RNA-Seq Analysis for Nucleus Genes Encoding Extensins in Arabidopsis. Genes were selected by >2-fold change with significant cut-off (yes or no) in at least one sample between 1 d and 7 d from RNA-Seq analysis, comparing Col-0 wild-type (WT) and *sig2-2* mutant. Classification of Arabidopsis Extensins was adapted from Cannon et al., 2008.

|  |  |  |  | 1 d | | | |  | 7 d | | | |
| --- | --- | --- | --- | --- | --- | --- | --- | --- | --- | --- | --- | --- |
| Group | Feature | Gene ID | AGI no. | WT | *sig2-2* | *sig2-2* /WT^a^ | cut-off^b^ |  | WT | *sig2-2* | *sig2-2* /WT^a^ | cut-off^b^ |
| I | Idt-poor | *EXT1* | *At1g76930* | 4 | 20 | 2.4 | yes |  | 164 | 622 | 1.9 | yes |
|  |  | *EXT23* | *At5g19810* | 1 | 0 | -1.1 | no |  | 1 | 1 | -0.2 | no |
|  |  |  |  |  |  |  |  |  |  |  |  |  |
| IIa | Idt-rich, | *EXT3* | *At1g21310* | 448 | 655 | 0.5 | yes |  | 753 | 528 | -0.5 | yes |
|  | Simple | *EXT18* | *At1g26250* | 0 | 0 | 0.6 | no |  | 29 | 14 | -1.0 | yes |
|  |  | *EXT19* | *At1g26240* | 0 | 0 | 0.0 | no |  | 4 | 1 | -1.6 | yes |
|  |  | *EXT20* | *At4g08370* | 0 | 0 | 0.0 | no |  | 0 | 0 | 0.0 | no |
|  |  | *EXT21* | *At2g43150* | 61 | 52 | -0.2 | no |  | 500 | 420 | -0.3 | yes |
|  |  | *EXT22* | *At4g08380* | 0 | 0 | 0.0 | no |  | 0 | 0 | 0.0 | no |
|  |  |  |  |  |  |  |  |  |  |  |  |  |
| IIb | Idt-rich, | *EXT6* | *At2g24980* | 14 | 123 | 3.2 | yes |  | 9 | 2 | -2.0 | yes |
|  | +SPSP | *EXT7* | *At4g08400* | 1 | 9 | 3.6 | yes |  | 2 | 0 | -1.8 | yes |
|  | motifs | *EXT8* | *At4g08410* | 3 | 29 | 3.3 | yes |  | 3 | 1 | -2.2 | yes |
|  |  | *EXT9* | *At5g06630* | 9 | 106 | 3.5 | yes |  | 7 | 1 | -2.4 | yes |
|  |  | *EXT10* | *At5g06640* | 12 | 119 | 3.3 | yes |  | 8 | 2 | -2.2 | yes |
|  |  | *EXT11* | *At5g49080* | 9 | 87 | 3.2 | yes |  | 5 | 1 | -2.4 | yes |
|  |  | *EXT12* | *At4g13390* | 6 | 67 | 3.4 | yes |  | 2 | 1 | -1.4 | yes |
|  |  | *EXT13* | *At5g35190* | 11 | 165 | 3.9 | yes |  | 17 | 4 | -2.2 | yes |
|  |  |  |  |  |  |  |  |  |  |  |  |  |
| IIc | Idt-rich, | *EXT2* | *At3g54590* | 14 | 124 | 3.1 | yes |  | 18 | 7 | -1.4 | yes |
|  | +SPSP | *EXT15* | *At1g23720* | 39 | 242 | 2.6 | yes |  | 97 | 99 | 0.0 | no |
|  | + a Cys | *EXT16* | *At3g28550* | 39 | 218 | 2.5 | yes |  | 61 | 56 | -0.1 | no |
|  | motif | *EXT17* | *At3g54580* | 19 | 140 | 2.9 | yes |  | 31 | 15 | -1.0 | yes |

^a^ fold-change (in log2 scale)

^b^ significant with q-value < 0.05.

**Supplemental Table 5.** RNA-Seq Analysis for GA-Biosynthesis and Catabolism Genes. Genes were as identified in Yamaguchi, S. 2006.

|  | |  |  | 1 d | | | | | |  | 7 d | | | |
| --- | --- | --- | --- | --- | --- | --- | --- | --- | --- | --- | --- | --- | --- | --- |
| AGI no. | Gene ID | | Another common name | WT | | *sig2-2* | *sig2-2* /WT^a^ | cut-off^b^ |  | | WT | *sig2-2* | *sig2-2* /WT^a^ | cut-off^b^ |
| *At4g02780* | *AtCPS* | | *GA1* | | 0.17 | 0.11 | -0.60 | no |  | | 0.23 | 0.24 | 0.05 | no |
| *At1g79460* | *AtKS* | | *GA2* | | 3.62 | 3.85 | 0.09 | no |  | | 5.15 | 4.39 | -0.23 | no |
| *At5g25900* | *AtKO* | | *GA3* | | 28.89 | 29.61 | 0.04 | no |  | | 23.19 | 19.28 | -0.27 | yes |
| *At1g05160* | *AtKAO1* | | *CYP88A3* | | 12.89 | 11.38 | -0.18 | no |  | | 6.24 | 8.45 | 0.44 | yes |
| *At2g32440* | *AtKAO2* | | *KAO2* | | 25.75 | 22.28 | -0.21 | no |  | | 14.68 | 19.03 | 0.37 | yes |
| *At4g25420* | *AtGA20ox1* | | *GA5* | | 2.45 | 3.42 | 0.48 | no |  | | 1.16 | 0.29 | -1.98 | no |
| *At5g51810* | *AtGA20ox2* | | *GA20OX2* | | 1.93 | 1.91 | -0.02 | no |  | | 1.09 | 1.57 | 0.52 | no |
| *At5g07200* | *AtGA20ox3* | | *YAP169* | | 1.31 | 0.73 | -0.83 | no |  | | 0.08 | 0.06 | -0.40 | no |
| *At1g60980* | *AtGA20ox4* | | *ATGA20OX4* | | 0.00 | 0.00 | 0.00 | no |  | | 0.04 | 0.00 | N/A | no |
| *At1g44090* | *AtGA20ox5* | | *ATGA20OX5* | | 0.00 | 0.15 | N/A | no |  | | 0.00 | 0.00 | 0.00 | no |
| *At1g15550* | *AtGA3ox1* | | *GA4* | | 20.49 | 25.51 | 0.32 | no |  | | 7.65 | 2.39 | -1.68 | yes |
| *At1g80340* | *AtGA3ox2* | | *GA4H* | | 67.56 | 69.10 | 0.03 | no |  | | 2.43 | 0.97 | -1.32 | yes |
| *At4g21690* | *AtGA3ox3* | | *ATGA3OX3* | | 0.00 | 0.00 | 0.00 | no |  | | 0.00 | 0.00 | 0.00 | no |
| *At1g80330* | *AtGA3ox4* | | *ATGA3OX4* | | 7.63 | 5.50 | -0.47 | no |  | | 2.61 | 3.52 | 0.43 | no |
| *At1g78440* | *AtGA2ox1* | | *ATGA2OX1* | | 0.11 | 0.26 | 1.29 | no |  | | 0.86 | 0.31 | -1.48 | no |
| *At1g30040* | *AtGA2ox2* | | *ATGA2OX2* | | 3.16 | 2.67 | -0.24 | no |  | | 2.88 | 2.99 | 0.06 | no |
| *At2g34555* | *AtGA2ox3* | | *ATGA2OX3* | | 0.00 | 0.00 | 0.00 | no |  | | 0.00 | 0.00 | 0.00 | no |
| *At1g47990* | *AtGA2ox4* | | *AtGA2ox4* | | 10.19 | 10.65 | 0.06 | no |  | | 18.01 | 10.66 | -0.76 | yes |
| *At3g17203* | *AtGA2ox5* | | *N/A* | | N/A | N/A | N/A | N/A |  | | N/A | N/A | N/A | N/A |
| *At1g02400* | *AtGA2ox6* | | *ATGA2OX4* | | 0.56 | 2.15 | 1.95 | no | |  | 4.43 | 4.45 | 0.01 | no |
| *At1g50960* | *AtGA2ox7* | | *ATGA2OX7* | | 0.00 | 0.00 | 0.00 | no | |  | 0.06 | 0.00 | N/A | no |
| *At4g21200* | *AtGA2ox8* | | *ATGA2OX8* | | 0.42 | 0.36 | -0.21 | no | |  | 0.89 | 0.61 | -0.53 | no |

^a^ fold-change (in log2 scale)

^b^ significant with q-value < 0.05.
