# Supplementary material for: Transcriptome and phenotyping analyses support a role for chloroplast sigma factor 2 in red‐light‐dependent regulation of growth, stress, and photosynthesis
Source: Plant Direct. 2018 Feb 19;2(2):e00043. doi: 10.1002/pld3.43 (PMC6508532; doi:10.1002/pld3.43)
Supplement: Supplementary file 2 [file PLD3-2-e00043-s002.pdf]

RESPONSE TO REVIEWERS –

Reviewer #1

It has been widely accepted that the nucleus and plastids communicate through anterograde (nucleus-to-plastid) and retrograde (plastid-to-nucleus) signaling pathways to coordinate nuclear and plastidial gene expression in a range of developmental and physiological processes. The authors have shown previously that SIG2 - a light-dependent sigma factor for the plastid-encoded RNA polymerase - is required for light-dependent growth and development by the red and far-red photoreceptors, the phytochromes. In this study, the authors expanded the analysis of the effects of SIG2 by performing transcriptome analysis of two development stages: 1d and 7d old seedlings. The results of these experiments reveal a link between SIG2 and the regulation of the plant growth hormone GA and stress responses by hydrogen peroxide.

Overall, this comprehensive study of the sig2 mutant provides new evidence that draws novel lines from plastidial gene expression to GA biogenesis and hydrogen peroxide responses. These results will be of interests to a general audience in plant biology, including people working on light and hormone signaling, oxidative stress, and plastid development. My only comment is that the authors might want to revise the manuscript, particularly the subtitles, to avoid the misunderstanding that SIG2 directly regulates the nuclear transcriptomic responses. The current model, as the authors stated, is that the activity of the plastid-encoded RNA polymerase sends a signal through the GUN signaling to impact nuclear gene expression. But some of the subtitles might leave the readers an impression that SIG2 plays a direct role in transcriptional regulation of the nuclear genes or nuclear function. For example: "SIG2 is required for the transcriptional regulation of a distinct group of nuclear genes, including ...", "Role of SIG2 in GA-signaling during photomorphogenesis", "Transcriptional role of SIG2 in H2O2-mediated stress response", and "SIG2 is required for the regulation of photosynthesis-related genes ...".

RESPONSE: In response to this helpful suggestion of the reviewer, subtitles in “Results” and “Discussion” sections have been edited for clarity as detailed below:

“SIG2 is required for the transcriptional regulation of a distinct group of nuclear genes, including growth-, GA-, stress-, and photosynthesis-related genes” edited to “SIG2 is required for the retrograde signaling-dependent transcriptional regulation of a distinct group of nuclear genes, including growth-, GA-, stress-, and photosynthesis-related genes”

“Role of SIG2 in GA-signaling during photomorphogenesis” edited to “Retrograde-dependent role of SIG2 in GA-signaling during photomorphogenesis

“Transcriptional role of SIG2 in H_2O_2-mediated stress response” edited to “Retrograde-associated transcriptional role of SIG2 in H_2O_2-mediated stress response”

“SIG2 is required for the regulation of photosynthesis-related genes and maintenance of photosynthetic efficiency” edited to “SIG2 is required for the retrograde signaling-dependent regulation of photosynthesis-related genes in red light and maintenance of photosynthetic efficiency”

“SIG2 contributes the regulation of photosynthesis-related genes” edited to “SIG2 contributes to the retrograde signaling-dependent regulation of photosynthesis-related genes in red light”

Additional text changes that have also been made for clarity are as follows:

Pg. 9, lines 245-247: “Our RNA-Seq data, altered sensitivity to GA3, and standard responsiveness to PAC in sig2 mutants suggested a transcriptional role of SIG2 in GA-dependent processes during photomorphogenesis.” edited to “Our RNA-Seq data for GA signaling-related genes, altered *sig2* mutant sensitivity to GA3, and standard responsiveness to PAC in sig2 mutants suggested a retrograde-associated transcriptional role of SIG2 in GA-dependent processes during photomorphogenesis.”

Pg. 15, lines 438-439: “Functional analysis of genes misregulated in sig2-2 under R light conditions suggested a potential role of SIG2 in GA homeostasis or signaling:” edited to

“Functional analysis of genes misregulated in sig2-2 under R light conditions suggested a potential retrograde signaling-dependent role of SIG2 in GA homeostasis or signaling:”

Pg. 17, lines 501-503: “Our functional classification from RNA-seq analysis suggested that SIG2 is required for the regulation of photosynthesis-related genes in red light (Figures 1B and 1D) as expected based on prior analyses (Woodson et al., 2013).” edited to “Our functional classification from RNA-seq analysis suggested that SIG2 is required for the retrograde-associated regulation of photosynthesis-related genes in red light (Figures 1B and 1D) as expected based on prior analyses (Woodson et al., 2013).”

Reviewer #2

This manuscript provides an extensive physiological and molecular study of an important area of plant photomorphogenesis. Through a series of elegant experiments, the authors demonstrated that SIG2 regulates a broader range of physiological responses in plants, including a specific role in red-light mediated photomorphogenesis. They also show that SIG2 plays important roles in red-light dependent photomorphogenic development and photosynthesis.

The experiments are comprehensive, well-designed, and provide new insights into light regulation in plants. The paper is sound, and the presentation is very good. The figures and tables are appropriate. The results support the conclusions. I do have some suggestions for improvements.

1. Major suggestion. I recommend that the authors do an experiment to examine red-light-dependent phototropism in roots. They could compare red-light root phototropism, which is a weak tropism, in the sig2 mutant and WT. There might be a role here as the sig2 mutant shows some of the defects found in the phyB mutant (which has been shown to be reduced in red-light phototropism). These experiments would be relatively simple and quick. Potentially, these experiments could expand the known role of SIG2 into regulation of tropisms. You can consult these two papers (which should be cited): Plant Physiology 131:1411-1417 (2003); Planta 236:635-645 (2012).

RESPONSE: Given the prior observation of red-light-dependent phototropism in roots and an association of phyA and especially phyB activity in regulating this process, the suggestion of the reviewer was intriguing. While aware of the literature suggested, we carefully reviewed the protocols and phenotypes observed in red-light-dependent root phototropic assays. As indicated by the reviewer, red-light-dependent phototropism in Arabidopsis roots is a weak tropism – particularly in standard growth conditions and in wild-type seedlings. While some increase in phototropism is observed in mutants, and in wild-type under microgravity conditions or using a digital rotating stage with feedback system, we determined that setting up experimental conditions to adequately conduct these experiments (for which we do not currently have a rotating stage setup or an ability to conduct microgravity-like growth) extends beyond the scope of the current investigation given the weak tropism phenotype. These are certainly reasonable experimental suggestions for a future focus.

2. Methods. What is the nature of the red light used? Was it from LEDs? If so, what are the wavelengths and catalog numbers? This is an important detail in photomorphogensis experiments.

RESPONSE: We have edited the section in the Experimental Procedures to reflect the nature, manufacturer and wavelength of light used in the red light growth. Specifically, "Seeds on solid medium were stratified for four days at 4°C in the dark and were then incubated in a Percival model E-30LED growth chamber (Percival) with constant red (Rc) illumination for one (1 d) or seven days (7 d) at 22°C." has been edited to "Seeds on solid medium were stratified for four days at 4°C in the dark and were then incubated in a Percival LED (light-emitting diode)-equipped growth chamber (Model: E-30LED, Serial Number: 6447.02.04J) with red (R) LEDs (λmax ~670 nm) under constant red (Rc) illumination for one (1 d) or seven days (7 d) at 22°C."

3. Methods. Provide some more details on the statistical methods. What type of software was used?

RESPONSE: In response to this suggestion of the reviewer, the following was added to the "EXPERIMENTAL PROCEDURES" section:

Statistical analyses

For statistical analysis, two-tailed Student's t-test (unpaired with two-sample equal variance) was performed with Microsoft Excel 2007 software.

4. Methods. Whenever % is used, indicate w/w, w/v, or v/v. This is done in some places but not in others.

RESPONSE:

We have edited the following passages in the Experimental Procedures in response to this reviewer's comment –

"with 80 % of acetone and chlorophyll concentrations"…changed to "80% (v/v)"

"…..leaf tissue with 80% acetone and quantified according"… changed to "80% (v/v)"

"….. Proteins were resolved on 15% SDS-PAGE gel and"...changed to "15% (w/v)"

"….. The membrane was blocked with 2 % bovine serum albumin"…changed to "2% (w/v)"
